# Profiling of Selected MicroRNAs in Proliferative Eutopic Endometrium of Women with Ovarian Endometriosis

**DOI:** 10.1155/2015/760698

**Published:** 2015-08-20

**Authors:** P. Laudanski, R. Charkiewicz, A. Tolwinska, J. Szamatowicz, A. Charkiewicz, J. Niklinski

**Affiliations:** ^1^Department of Perinatology, Medical University of Bialystok, Ulica Marii Sklodowskiej-Curie 24a, 15-276 Bialystok, Poland; ^2^Department of Clinical Molecular Biology, Medical University of Bialystok, Ul. Waszyngtona 13, 15-269 Bialystok, Poland; ^3^Clinic “EDMED” Białystok, Ul. Piasta 14, 15-044 Bialystok, Poland; ^4^Department of Gynecology and Gynecological Oncology, Medical University of Bialystok, Ulica Marii Sklodowskiej-Curie 24a, 15-276 Bialystok, Poland; ^5^Department of Medical Pathomorphology, Medical University of Bialystok, Ul. Waszyngtona 13, 15-269 Bialystok, Poland

## Abstract

It has been well documented that aberrant expression of selected microRNAs (miRNAs) might contribute to the pathogenesis of disease. The aim of the present study is to compare miRNA expression by the most comprehensive locked-nucleic acid (LNA) miRNA microarray in eutopic endometrium of patients with endometriosis and control. In the study we recruited 21 patients with endometriosis and 25 were disease-free women. The miRNA expression profiles were determined using the LNA miRNA microarray and validated for selected molecules by real-time PCR. We identified 1198 human miRNAs significantly differentially altered in endometriosis versus control samples using false discovery rate of <5%. However only 136 miRNAs showed differential regulation by fold change of at least 1.3. By the use of selected statistical analysis we obtained 45 potential pathways that might play a role in the pathogenesis of endometriosis. We also found that natural killer cell mediated cytotoxicity pathway was found to be inhibited which is consistent with previous studies. There are several pathways that may be potentially dysregulated, due to abnormal miRNA expression, in eutopic endometrium of patients with endometriosis and in this way contribute to its pathogenesis.

## 1. Introduction

Endometriosis is relatively common, benign gynecological disease in which endometrial tissue ectopically implants in a location outside the uterine cavity. The most common clinical symptoms include pelvic pain and infertility which can seriously influence the quality of life of affected patients [1].

Despite the current controversy regarding the pathophysiology of this disease, Sampson's theory explains the existence of endometrial cells in the peritoneal cavity by retrograde menstruation. Several factors such as increased chemokines and matrix metalloproteinases in the peritoneal fluid (PF), angiogenesis, tumor suppressor, and oncogenes as well as upregulating of proinflammatory cytokines may facilitate the pathogenesis of endometriosis [2–6]. It is therefore assumed to be a complex process, in many aspects characteristic of systemic disease. It has now also become widely accepted that molecular aberration within the eutopic endometrium may predispose a subgroup of women to the development of endometriosis [7].

The molecular changes that could potentially lead to abnormal growth of endometrium outside uterus include microRNAs (miRNAs) expression fluctuations [8]. MicroRNAs are a large class of endogenous, single-stranded, and short ncRNA (noncoding) of approximately 22 nucleotides in length that play a key role in regulating gene expression through interaction with mRNA of protein-coding genes [9]. miRNA expression is tissue- and cell-specific and it was demonstrated that miRNAs are important in endometriosis and associated reproductive conditions [10].

In our recent study we also showed that, out of 667 miRNAs, two molecules, namely, hsa-miR-483-5p and hsa-miR-629^*∗*^, are significantly downregulated in eutopic endometrium of patients with ovarian endometriosis, which could be a consequence of an early defect in the physiological activity of the proliferative endometrium [11]. Since there have been only few studies, in the available literature, which concerned miRNA profiling of eutopic endometrium (actually mostly luteal) [12, 13], and the technology since our last publication has greatly developed, we decided to expand our previous work and use more robust microarray technique which facilitates expression profiling of more than 2000 miRNAs. The aim of the present study is to examine possible differential regulation as well as putative pathways that might be regulated by abnormal miRNA expression in the proliferative eutopic endometrium of patients with advanced ovarian endometriosis.

## 2. Material and Methods

Patients (*n* = 46) scheduled for laparoscopy for adnexal mass or infertility at the Medical University of Bialystok were recruited to participate in this study. Endometrial biopsies were collected using Pipelle suction curettes. Endometrial tissue samples were classified by histological dating according to the method of Noyes et al. [14] and only patients in the proliferative phase (days from 6th to 13th) of the cycle were included in the study.

Patients with endometriosis (Group I, *n* = 21) stages from III to IV were diagnosed by laparoscopic findings according to the revised American Fertility Society classification of endometriosis [15] and each case was confirmed by histopathology. As a control (Group II, *n* = 25) we used endometrial tissue from patients without any endometriosis visible during laparoscopy.

All women had regular menstrual cycles (28–30 days) and were not taking any medication for at least 3 months prior to operation. We excluded patients with autoimmune disease, pelvic inflammatory disease, adenomyosis, fibroids, and dysfunctional uterine bleeding. The study was approved by the Institutional Review Board of Medical University of Bialystok and informed consent was obtained from each patient.

The collected tissue was placed separately in buffered formalin for histopathological studies and in RNA later (Sigma-Aldrich, Poland) for molecular analysis. The latter was stored for 24 hours in +4°C and then tissues were transferred and stored in −80°C. Total RNA was extracted using the* mir*Vana miRNA Isolation Kit (Ambion, Life Technologies, Poland). RNA quality was assessed with Agilent Bioanalyzer 2100 and Agilent RNA 6000 Nano kit (Agilent Technologies, Perlan, Poland) and samples chosen for further analysis showed minimum sign of degradation as judged by the RNA integrity number (RIN), which was above 9 for all samples. RNA concentrations were measured on a NanoDrop 2000c (Thermo Scientific, Biotech, Poland).

### 2.1. miRNA Expression Profiling Using Exiqon Microarrays

For the purpose of miRNA microarray screening we chose 10 samples of patients with advanced endometriosis and 11 controls. Microarray analysis was conducted as single-channel Hy3 experiments on Exiqon's miRCURY LNA (locked-nucleic acid) microRNA Array 7th generation—hsa, mmu and rno. Exiqon arrays contain 3100 capture probes, complementary to most human, mouse, rat, and their related viral sequences from the v.19.0 release of miRBase. The arrays also contain 25 proprietary human miRPlus sequences not yet in miRBase. 500 ng RNA sample was labelled with a Hy3 fluorophore (Exiqon, Denmark). Labelling reactions were performed using Exiqon's miRCURY LNA microRNA Hi-Power Labeling Kit with the use of synthetic spike controls, Spike-in microRNA Kit v2 (Exiqon, Denmark), according to the manufacturer's protocol. Hybridization of labeled RNA to the array was performed in SureHyb chambers (Agilent Technologies, USA) for 16 hours at 56°C. Slides were washed according to manufacturer's instructions and scanned at 10 *μ*m resolution using an Agilent G2505C DNA Microarray Scanner. Raw data were generated using Imagene 9.0 software (BioDiscovery, Inc., USA), using an FE protocol available on demand from Exiqon.

### 2.2. Quantitative PCR, Real-Time RT-PCR (qPCR)

The expression levels of the selected microRNAs and the assay miRBase IDs (miRBase Accession Number) are hsa-miR-4714-5p (MIMAT0019822), hsa-miR-4284 (MIMAT0016915), hsa-miR-5193 (MIMAT0021124), hsa-miR-4454 (MIMAT0018976), hsa-miR-3680-5p (MIMAT0018106), hsa-miR-3667-5p (MIMAT0018089), hsa-miR-23a-3p (MIMAT0000078), hsa-miR-23b-3p (MIMAT0000418), hsa-miR-5187-3p (MIMAT0021118), hsa-miR-3152-5p (MIMAT0019207), and hsa-miR-30d-5p (MIMAT0000245) which were evaluated using the miRCURY LNA Universal RT microRNA PCR system (Exiqon, Denmark). In the first step, we have conducted the one first-strand cDNA synthesis reaction, which provided template for all microRNA real-time PCR assays. The cDNA for each RNA sample was obtained using the Universal cDNA Synthesis Kit II (Exiqon, Denmark) according to the manufacturer's instructions. Levels of miRNAs were quantitated using individual miRCURY LNA Universal RT microRNA PCR Assays (Exiqon, Denmark).

The conditions for qPCR were as follows: 95°C for 10 min and 45 cycles of 95°C for 10 sec followed by 60°C for 1 min. Finally a melting curve analysis was performed with denaturation at 95°C for 15 s and 60°C for 15 s followed by a temperature gradient from 60 to 95°C for 20 min and a final denaturation at 95°C for 15 s. U6 snRNA was used as the endogenous control. LNA PCR amplification reactions for each miRNA molecule were repeated independently three times. Quantitative real-time PCR analysis was performed using the Applied Biosystems 7900HT System (Life Technologies, Foster City, CA).

Gene expression values were calculated based on the 2(−Delta  Delta  C(T)) method, where one sample was designated the calibrator, through which all other samples were analyzed [16]. Briefly, ΔCT represents the threshold cycle of the target minus that of U6 snRNA and ΔΔCT represents the ΔCT of each target minus that of the calibrator. Relative quantities were determined using the equation: RQ = 2^−ΔΔCT^. For the calibrator sample, that is, control RNA from eutopic endometrium, the equation is relative quantity = 2^−0^, which is 1; therefore, every other sample is expressed relative to this.

### 2.3. Statistical Analysis

All data analyses were performed in R statistical environment (http://www.r-project.org/) and relevant Bioconductor software [17].

The raw microarray data were preprocessed with* vsn*2() function implemented in* vsn* package [18]. The* vsn* function was used with default settings. Differentially expressed miRNAs were identified with* limma* [19]. In order to associate miRNA with mRNA we used annotations from 6 databases: miRBase, targetScan, miRanda, tarBase, mirTarget2, and picTar. Probes not associated with human mRNA were not analyzed further. The potential influence of miRNA on mRNA expression was visualized using signaling pathway definitions.

In our study we used signaling pathway definitions from KEGG (*Kyoto Encyclopedia of Genes and Genomes* [20]). The potential miRNA influence on mRNA expression was also used to analyze GO (gene ontology) terms potentially overrepresented in disturbed genes.

To assess perturbation of signaling pathways we applied SPIA (*Signaling Pathway Impact Analysis* [21]). We used the potential influence of miRNA on mRNA expression instead of gene expression data.

While comparing two groups for quantitative data, Mann-Whitney-Wilcoxon test was used due to the nonnormal distribution of the tested variables. The significance level was equal to 0.05. The calculations have been carried out by means of Microsoft Excel spreadsheet and STATISTICA, StatSoft, Inc. Version 7.1. statistical package (data analysis software system).

## 3. Results

Patients clinical characteristics are presented in Table 1.

We identified 1198 human miRNAs significantly differentially altered in endometriosis versus control samples using false discovery rate of <5% (Table I supplement in the supplementary Material available online at http://dx.doi.org/10.1155/2015/760698).

**Figure 1:**
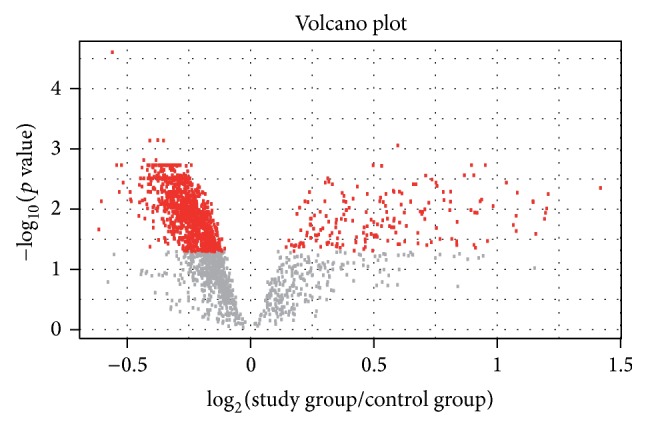
Volcano plot for all analyzed human miRNAs. The *x*-axis (horizontal) is the fold change between endometriosis and control samples (on a log scale, so that up- and downregulation appear symmetric), and *y*-axis represents the *p* value for a test of differences between samples (most conveniently on a negative log scale, so smaller *p* values appear higher up). Red dots represent miRNAs significantly differentially expressed.

**Figure 2:**
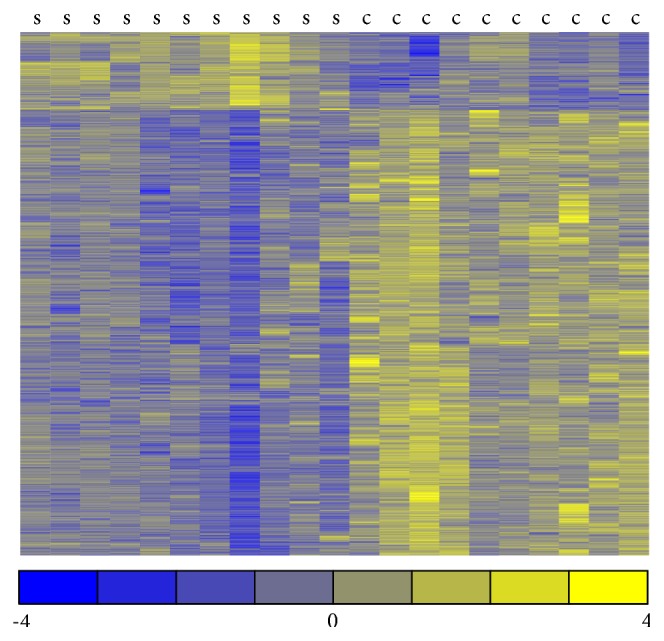
Heatmap display of significantly expressed miRNAs. Heatmap representation of expression data for miRNAs significantly altered in endometriosis samples compared with control samples (adjusted *p* value < 0.05). Columns correspond to samples and rows correspond to individual miRNAs. For a given miRNA an average value was computed and subtracted from each observation. The yellow color marks a higher expression (over average expression in two groups), while the blue color represents a lower expression (again with regard to average expression). The color scale represents the magnitude of changes. “s” stands for endometriosis samples and “c” stands for control samples.

Volcano plot (Figure 1) presents results of expression analysis for all analyzed miRNAs, while the heatmap (Figure 2) shows expression profiles only of significantly expressed miRNAs.

The associations between miRNA and mRNA are presented in the zipped supplement directory (called Table II supplement).

**Figure 3:**
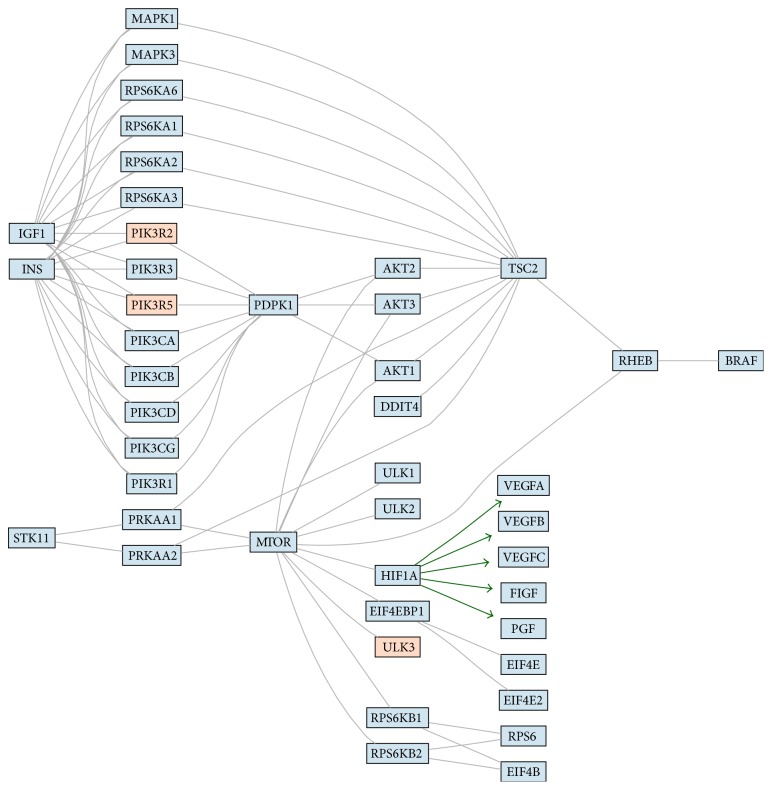
The figure shows signaling pathways (3: mTOR and 4: VEGF) with marked hypothetical influence of miRNA. The colors present expression of miRNAs assigned to a particular gene. The blue color stands for higher expression in control samples, and the red color stands for higher expression endometriosis. The color scale represents the magnitude of changes. The green arrows represent connections start from transcription factor and which hypothetical expression changes are consistent with hypothetical expression changes of genes regulated by this transcription factor.

**Figure 4:**
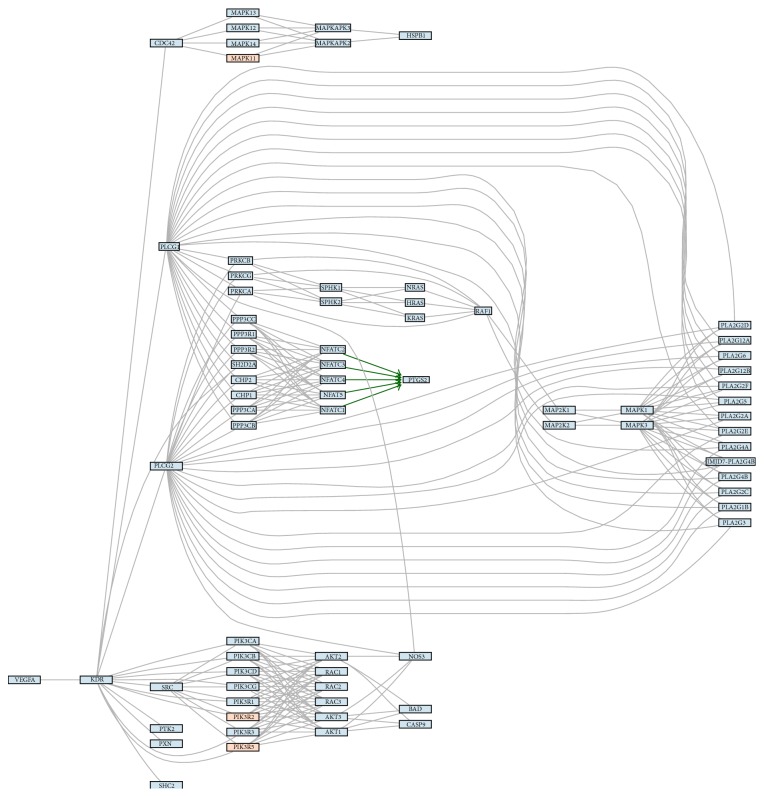
The figure shows signaling pathways (3: mTOR and 4: VEGF) with marked hypothetical influence of miRNA. The colors present expression of miRNAs assigned to a particular gene. The blue color stands for higher expression in control samples, and the red color stands for higher expression endometriosis. The color scale represents the magnitude of changes. The green arrows represent connections start from transcription factor and which hypothetical expression changes are consistent with hypothetical expression changes of genes regulated by this transcription factor.

We obtained 45 potential pathways from the KEGG (Kyoto Encyclopedia of Genes and Genomes) database and particularly mTOR and VEGF signaling pathway caught our attention due to its close potential relation to pathogenesis of endometriosis (Figures 3 and 4, resp.).

The significantly overexpressed GO (gene ontology) terms are presented in Table 2. We found six potential cellular processes that involve protein synthesis to be potentially regulated by influence of miRNA on mRNA, including translational elongation and termination, protein targeting and localization to endoplasmic reticulum, cotranslational protein targeting to membrane, SRP- (signal-recognition particle-) dependent cotranslational protein targeting to membrane, and establishment of protein localization to endoplasmic reticulum.

The SPIA showed perturbation of potentially nine pathways in two analyzed groups. Most interestingly natural killer cell mediated cytotoxicity pathway was found to be inhibited in eutopic endometrium of patients with endometriosis as compared with control (Table 3).

### 3.1. Validation by RT-PCR

Following the selection of miRNAs by fold change filtering (fold change > 1.3), we found that there were 136 upregulated miRNAs and no downregulated miRNAs in the eutopic endometrium of patients with advanced ovarian endometriosis compared with the eutopic endometrium.

We then validated 11 selected miRNAs but we were not able to observe clear statistical differences as to the expression of any of the chosen molecule (Table 4) between studied groups. We found that, specifically for three miRNAs, that is, miR-5187-3p, miR-3152-5p, and miR-30d-5p, there exist differences with border significance within 0.05 (Table 4).

## 4. Discussion

Although in our study we found more than 1000 miRNAs that are significantly differentially regulated between eutopic endometrium of patients with and without endometriosis, the differences, either up- or downregulated, were not higher than 2.6-fold in any case. The problem with obtaining clearly significant qPCR results may point to reservations that we may have as to the expression array results which can indirectly imply that some of the results might be false positives and that there are a number of variables that must also be considered. However, we must emphasize that although qPCR is the method of choice of confirming gene expression changes, the current technology contains a vital limitation related to experiments of this nature [22]. Specifically, it is not sensitive enough to accurately detect low, but statistically significant, fold changes of around 2-fold [23, 24] that are typical for miRNAs [25, 26]. It is more and more accepted that with the advent of new quantitative digital PCR technologies it is much more likely to detect small miRNA differences [27, 28]. Interestingly we found that for 3 validated miRNAs it followed the same trend, up or down, similar to the array results.

Our study showed little concordance with the endometriosis-associated miRNAs identified by two previous published studies, which also had minimal convergence with one another.

In the first miRNA eutopic versus normal endometriosis study, Pan et al. profiled the expression of 287 miRNAs in paired early-mid secretory eutopic and ectopic endometrium and isolated endometrial cells from women with stage III endometriosis and compared to control by the use of mirVana miRNA Bioarray. They also aimed to compare the expression of selected miRNA between isolated endometrial stromal cell (ESC) and glandular epithelial cell (GEC).

It was found that 65 of these miRNAs were identified to be expressed above the threshold levels set during the analysis in the endometrium of women without endometriosis. By the use of ANOVA it was also identified that there are 48 miRNAs which are differentially expressed between different combinations of eutopic and ectopic endometria. Specifically it was observed that there exists a progressive decline in miRNAs numbers from endometrium of women without endometriosis to eutopic endometrium, ectopic endometrium, and ectopic endometrium without paired eutopic tissue (miR23a and miR23b). It is somewhat confusing since it shows that there might be potential differences between ectopic endometrium of patients in whom eutopic endometrium was collected as compared with patients where this procedure was not performed. It was also shown that 32 miRNAs are differentially expressed in ESC and GEC, a significantly lower numbers when compared with endometrium of women without endometriosis. The obvious limitation of the above study was very limited sample size, a total of 16 samples including those with and without endometriosis as well as those without paired eutopic tissue, and restriction to only second phase of the cycle [13].

On the other hand, it was also more recently shown in advanced (III-IV) disease that miR23a and miR23b were significantly decreased in proliferative ectopic and eutopic endometrium compared with normal endometrium [29]. It was one of the reasons to choose above two molecules in our validation phase of the study; nonetheless were not able to observe any significant differences between eutopic endometria of patients with and without endometriosis.

It was also shown that in the early secretory endometrium (ESE), by the use of LNA microarray (which consisted of Tm-normalized capture probes for 1488 distinct miRNAs), there are only 6 miRNAs which were differentially expressed with a fold change of >1.5 in the ESE from women with versus without endometriosis [12]. Downregulated miRNAs included miR-9, miR-9^*∗*^, miR-34b^*∗*^, miR-34c-5p, miR-34c-3p, and the unannotated miRPlus_42 780. miR-9 represented the most significantly dysregulated miRNA. The majority of miRNAs were unchanged or not expressed in endometrium, in agreement with the previous data demonstrating spatiotemporal-specific expression of a high percentage of miRNAs. The validation by qRT-PCR showed that the trends for downregulation of miRNA expression were consistent in all four qRT-PCR measurements (that were chosen for validation) and significant for three of the four miRNAs. Interestingly miR-9^*∗*^ (5′-end form), as opposed to miR-9 (3′-end form), did not demonstrate statistically significant difference in expression between ESE from women with versus without endometriosis.

The above study is strengthened by the stringency of the surgical confirmation of presence or absence of disease and by the inclusion of only biopsy confirmed, moderate-severe (rAFS III-IV) stage endometriosis among the affected cohort. On the other hand, our study is strengthened by the use of microarray technology employing locked-nucleic acid (LNA) probes. Mature miRNAs are approximately 22 nucleotides in length, and this short length presents problems of specificity in their detection and localization. Microarray analysis using LNA probes allows very sensitive detection of these short-coding sequences [30].

Only very recently there have been published two studies which yielded quite different results. Shi et al. studied endometriotic, eutopic, and normal endometrial tissues in the proliferative phase of the cycle [31]. They used LNA microarrays which contained >1,700 capture probes covering miRNAs listed in miRBase v. 14.0. The selection of miRNAs was made by fold change >2 and it was found that, compared with the normal endometrium, 36 miRNAs were downregulated with no upregulated miRNAs in the eutopic endometrium of patients with endometriosis. Since, among these differentially expressed miRNAs, miR-183, miR-215, and miR-363 were found downregulated in both the ectopic and eutopic tissues, the authors selected miR-183 for validation of further functional studies. In our study we could not confirm differential expression of miR-183.

Second recent study concentrated on miRNA expression profile in relation to selected angiogenesis factors [32]. In their study GeneChip miRNA 2.0 Affymetrix array platform (that contains 1105 probes for mature human miRNAs) was used for screening and validation was performed by using LNA RT-PCR. The major limitation of this study is very low number of studied samples; that is, seven eutopic endometrial tissues and three ovarian endometrioma tissues were compared to five endometrial tissues from healthy controls. When the three sample categories were compared, they found 156 mature miRNAs that were differentially expressed at least 1.3-fold in ovarian endometrioma or in eutopic endometrium or in both tissues compared with healthy tissue. The results are quite surprising since it looks like there are 156 miRNAs which are common for ovarian endometrioma and eutopic endometrium from the same patients and this is significantly different from eutopic control endometrium. It is generally accepted that histological structure and biology of eutopic and ectopic endometria are generally different and it is highly unlikely that there are exactly 156 miRNAs which would have the same coexpression pattern. On the other hand, it is stated in the same results section that supervised hierarchical clustering of differentially expressed miRNAs showed similar patterns in control and eutopic endometrium, with ovarian endometrioma clustering separately from control and eutopic endometrium. And in this case (as opposed to 156 profiles) it would be logical since eutopic and ectopic endometria should substantially differ in gene expression profile.

In our own previous study we used different technique, based on TaqMan miRNA microfluidic cards and validated by TaqMan real-time PCR, and we found in similar samples that out of 667 miRNAs there are eventually two, that is, hsa-miR-483-5p and hsa-miR-629^*∗*^, which are significantly downregulated in patients with endometriosis [11]. In the present study we used different methodology and could not observe significant difference as to any of the above cited molecules between studied groups. The differences are not actually surprising in view of the most recent and comprehensive study by Mestdagh et al. where they evaluated quantitative miRNA expression platforms in the microRNA quality control and systematically compared 12 commercially platforms, including all microarrays (also TaqMan and LNA Exiqon). They observed substantial interplatform differences when evaluating differential miRNA expression with an average validation rate of only 54.6% for differentially expressed miRNAs. One of the most unexpected findings, of this enormous comparative study, was low concordance of differential expression [33].

Our* in silico* analysis, based on KEGG database (resource that integrates genomic, chemical, and systemic functional information) showed that there are potentially several pathways dysregulated by differentially regulated miRNAs and some of them like mTOR (mammalian target of rapamycin) or VEGF (vascular endothelial growth factor) have been previously studied in endometriosis [3, 6, 34–36].

In one of our studies we found that out of 15 different mTOR related genes including* NF1, RHEB, mTOR, PTEN, TSC1, TSC2, KRAS, S6K1, TP53, EIF4E, LKB1, PIK3CA, BECN1, 4EBP1,* and* AKT1* there are at least 2, that is,* AKT1* and* 4EBP1,* which were found to be upregulated in eutopic endometrium of patients as compared with control [3]. More recently we also studied 84 angiogenesis-related genes, with different VEGF types, and found at least 5 to be differentially regulated in the eutopic endometrium [6].

On the other hand, the most interesting finding of our study comes from Signaling Pathway Impact Analysis which showed that out of nine potentially perturbed pathways in two analyzed groups it is natural killer cell mediated cytotoxicity which is inhibited in eutopic endometrium of patients with endometriosis. The inhibition of natural killer cells activity has been widely accepted [37, 38] in endometriosis; however, very few studies concentrated on the potential disturbances existing in the eutopic endometria of patients with endometriosis. As mentioned earlier our results can be partly complicated by additional limitations which have been previously discussed [3–6].

Briefly the problem may be the structure of control group, which is limited to infertile patients and simple ovarian cysts. It is therefore difficult to evaluate whether control group is a healthy one alone since infertility and cysts themselves might to some degree influence expression of genes in endometrium. We also think that the result could also represent the heterogenous nature of whole endometrium tissue which is composed of different cellular compartments which create the potential for a large amount of biological variation. On the other hand, we intentionally decided not to perform laser capture microdissection (LCM) since there are several reasons, described in the discussion section of the article by Borghese et al. [39], why this relatively modern histopathological technique is not necessarily feasible in the studies on endometriosis.

As we already discussed it previously in our publications [3–6] it is important to mention that in Poland it is impossible to collect the most suitable healthy control samples in endometriosis research, like those taken during sterilization, since it is an illegal form of contraception. On the other hand, it is also very difficult to evaluate whether even the most proper control group is a healthy one alone. It was shown that 6% of cases, where macroscopically normal pelvic anatomy was found, presented microscopic endometriotic lesions [40].

In summary, we found that there are at least 136 miRNAs with differential expression fold change of at least 1.3 that could be involved in the pathogenesis of endometriosis. It seems however that current quantitative PCR methods are not always sensitive enough to detect differences in the expression of miRNA of less than 2.5. It is hoped that with advent of digital PCR it will be possible to more effectively detect small miRNA expression differences which may have significant impact on the discovery of potential biomarkers. We also found that potential several pathways, including mTOR, VEGF, natural killer cell cytotoxicity, and at least 6 potential cellular processes, involved mainly in protein synthesis, may be regulated by abnormal miRNAs expression. The confirmation of their potential role requires further functional studies.

## 5. Conclusions

We identified several miRNAs and potentially new pathways that may be abnormally regulated in eutopic endometrium of patients with endometriosis which may contribute to the pathogenesis of this debilitating disease.

## Supplementary Material

Table I: The names of miRNAs significantly differentially expressed between eutopic endometrium of patients with endometriosis vs eutopic endometrium of patients without endometriosis.
Table II: The associations between miRNA and mRNA, which presents detailed results of the search of six major databases i.e targetscan, tarbase, miranda, mirbase, mirtarget2 and pictar.

## Figures and Tables

**Table 1:** Clinical characteristics of patients.

	Endometriosis (*n* = 21)	Control (*n* = 25)
Age (years)	31.35 ± 0.90	30.73 ± 0.91
Infertility, *n* (%)	9 (50)	16 (64)
Primary	7	8
Secondary	2	8
Duration of infertility (months)	48.2 ± 19.4 (23–71)	50.8 ± 15.8 (13–71)
Ovarian cysts, *n* (%)		
Endometrial	21 (100)	—
Simple	—	9 (36)

Data are mean ± SEM. Ranges are provided for “duration of infertility.”

**Table 2:** Gene ontology (GO) terms overrepresented in eutopic endometrium of patients with endometriosis and control.

GO term	Adjusted *p* values for overrepresentation analysis for miRNAs with higher expression in endometriosis	Adjusted *p* values for overrepresentation analysis for miRNAs with higher expression in control
Translational elongation	1	0.077

Translational termination	1	0.077

Cotranslational protein targeting to membrane	1	0.077

SRP- (signal-recognition particle-) dependent cotranslational protein targeting to membrane	1	0.077

Protein targeting to endoplasmic reticulum	1	0.077

Protein localization to endoplasmic reticulum	1	0.077

Establishment of protein localization to endoplasmic reticulum	1	0.077

The first column contains names of significantly overrepresented GO terms. Both the second and the third columns contain adjusted *p* values for overrepresentation analysis (Fisher's exact test). The second column presents results for genes associated with miRNA that have higher expression in endometriosis (of at least 20%) and the third column presents results for genes associated with miRNA that present higher expression in control samples (of at least 20%). GO terms with adjusted *p* values <0.25 were recognized as significantly overrepresented.

**Table 3:** Signaling Pathway Impact Analysis (SPIA) showing perturbation of signaling pathways in eutopic endometrium of patients with endometriosis versus control.

The name of the pathway	Adjusted *p* values	Status
Alcoholism	0	A
Olfactory_transduction	0	A
Viral_carcinogenesis	0	I
Systemic_lupus_erythematosus	0.01	A
Chronic_myeloid_leukemia	0.01	A
Cytosolic_DNA-sensing_pathway	0.01	A
Natural_killer_cell_mediated_cytotoxicity	0.01	I
RNA_transport	0.03	I
Neurotrophin_signaling_pathway	0.04	A

The first column shows names of significantly impacted pathways, the second column contains adjusted *p* values, and the third shows type of impact [activation (A)/inhibition (I)] in the analyzed group of samples with reference to the control group.

**Table 4:** Relative quantity (RQ) values of selected miRNA as validated by real-time PCR in patients with endometriosis (*n* = 42) and control (*n* = 25).

Number	miRNA	Endometriosis median (minimum–maximum values)	Control median (minimum–maximum values)	*p* value	Fold change difference between endometriosis and control by miRNA microarrays
1	miR-4454	0.34 (0.07–2.07)	0.41 (0.14–3.5)	0.34	1.51
2	miR-4714-5p	0.63 (0.05–9.9)	0.9 (0.01–4)	0.9	1.86
3	miR-5193	0.29 (0.04–11.3)	0.51 (0.04–22.9)	0.13	1.93
4	miR-4284	1.88 (0.1–10.3)	1.6 (0.2–15.7)	0.83	2.28
5	miR-5187-3p	0.28 (0.05–3.2)	0.20 (0.07–0.69)	0.051	1.41
6	miR-3680-5p	0.8 (0.07–31.6)	1.2 (0.06–11.2)	0.96	2.21
7	miR-3667-5p	0.68 (0.06–18.19)	1.8 (0.07–8.5)	0.63	2.3
8	miR-3152-5p	0.17 (0.03–1.4)	0.27 (0.002–2.8)	0.048	1.44
9	miR-23a-3p	1.22 (0.02–11.3)	1.17 (0.03–12.3)	0.48	1.55
10	miR-23b-3p	0.4 (0.06–1.7)	0.5 (0.1–2.2)	0.44	2.08
11	miR-30d-5p	0.22 (0.05–21)	0.42 (0.1–40)	0.05	1.37
