# Supplementary material for: Profiling of Selected MicroRNAs in Proliferative Eutopic Endometrium of Women with Ovarian Endometriosis
Source: Biomed Res Int. 2015 Aug 20;2015:760698. doi: 10.1155/2015/760698 (PMC4558423; doi:10.1155/2015/760698)
Supplement: Supplementary file 1 — Table I: The names of miRNAs significantly differentially expressed between eutopic endometrium of patients with endometriosis vs eutopic endometrium of patients without endometriosis. Table II: The associations between miRNA and mRNA, which presents detailed results of the search of six major databases i.e targetscan, tarbase, miranda, mirbase, mirtarget2 and pictar. [file 760698.f1.zip › 760698.f1/Table I.pdf]

Table I supplement

Table presents names of miRNAs significantly differentially expressed between eutopic endometrium of patients with endometriosis (study group) vs eutopic endometrium of patients without endometriosis (control). The columns contain miRNAs' ID, log-expression ratio (log2(endometriosis/control)) adjusted p-values and fold-change values, respectively.

ID	log2(endometriosis/control)	adj.P.Val	Fold change
hsa-miR-4717-3p	-0.557	0.002*	0.67971412
hsa-miR-3127-3p	-0.373	0.001	0.77217513
hsa-miR-5705	-0.406	0.001	0.75471298
hsa-miR-4667-3p	-0.35	0.001	0.7845841
hsa-miR-4454	0.6	0.001	1.51571657
hsa-miR-4515	-0.43	0.002	0.74226179
hsa-miR-4529-3p	-0.38	0.002	0.76843759
hsa-miR-5193	0.955	0.002	1.93857963
hsa-miR-548f	-0.325	0.002	0.79829839
hiv1-miR-TAR-3p	-0.345	0.002	0.78730798
hsa-miR-518a-3p	-0.348	0.002	0.78567252
hsa-miR-3157-5p	-0.52	0.002	0.69737183
hsa-miR-4527	-0.299	0.002	0.8128156
hsa-miR-632	-0.335	0.002	0.79278414
hsa-miR-4477a	-0.307	0.002	0.80832087
hsa-miR-3619-3p	-0.323	0.002	0.79940583
hsa-miR-3140-5p	-0.333	0.002	0.79388393
hsa-miR-4685-3p	-0.323	0.002	0.79940583
hsa-miR-378f	-0.379	0.002	0.76897042
hsa-miR-5703	-0.54	0.002	0.68777091
kshv-miR-K12-2-3p	-0.283	0.002	0.82188019
hsa-miR-548am-3p	-0.293	0.002	0.81620305
hsa-miR-566	-0.353	0.002	0.7829543
hsa-miR-1193	-0.409	0.002	0.75314523
hsa-miR-4683	-0.395	0.002	0.76048938
hsa-miR-3116	-0.384	0.002	0.76630998
hsa-miR-3912	-0.289	0.002	0.81846918
hsa-miR-3190-5p	-0.392	0.002	0.76207242
hsa-miR-3191-5p	-0.237	0.002	0.8485079
hsa-miR-410	-0.355	0.002	0.78186964
hsa-miR-4777-3p	-0.314	0.002	0.80440837
hsa-miR-770-5p	-0.297	0.002	0.81394319
hsa-miR-4714-5p	0.899	0.002	1.86477297
hsa-miR-4446-3p	-0.282	0.002	0.82245007
hsa-miR-4481	-0.414	0.002	0.75053955
hsa-miR-5187-3p	0.5	0.002	1.41421356
hsa-miR-3187-5p	-0.367	0.002	0.77539321
hsa-miR-4685-5p	-0.409	0.002	0.75314523
hsa-miR-525-5p	-0.363	0.002	0.77754604
hsa-miR-3173-5p	-0.338	0.002	0.7911373
hsa-miR-3152-5p	0.534	0.002	1.44793817
hsa-miR-518c-3p	-0.296	0.002	0.81450756
hsa-miR-26a-2-3p	-0.258	0.002	0.8362464
hsa-miR-5197-3p	-0.395	0.002	0.76048938

hsa-miR-4252	-0.323	0.002	0.79940583
hsa-miR-541-5p	-0.314	0.002	0.80440837
hsa-miR-1269b	-0.358	0.002	0.78024548
hsa-miR-1207-5p	-0.437	0.002	0.73866903
hsa-miR-4465	-0.392	0.002	0.76207242
hsa-miR-5011-3p	-0.323	0.002	0.79940583
hsa-miR-658	-0.309	0.002	0.80720107
hsa-miR-3155a	-0.415	0.002	0.75001949
hsa-miR-551b-5p	-0.347	0.002	0.78621729
hsa-miR-302f	-0.318	0.002	0.80218117
hsa-miR-654-5p	-0.353	0.002	0.7829543
hsa-miR-518c-5p	-0.252	0.002	0.83973149
hsa-miR-150-3p	-0.304	0.002	0.81000347
hsa-miR-4767	-0.384	0.002	0.76630998
hsa-miR-5706	-0.362	0.002	0.77808518
hsa-miR-5190	-0.371	0.003	0.77324634
hsa-miR-3974	-0.267	0.003	0.83104586
hsa-miR-4675	-0.407	0.003	0.75419004
hsa-miR-548a-3p	-0.345	0.003	0.78730798
hsa-miR-1248	0.87	0.003	1.8276629
hsa-miR-1273c	-0.294	0.003	0.81563749
hsa-miR-4475	0.908	0.003	1.87644239
hsa-miR-3122	-0.314	0.003	0.80440837
hsa-miR-583	-0.279	0.003	0.82416208
hsa-miR-302d-5p	-0.35	0.003	0.7845841
hsa-miR-3163	-0.286	0.003	0.82017291
hsa-miR-92a-3p	0.713	0.003	1.63920922
hsa-miR-181a-3p	-0.286	0.003	0.82017291
hsa-miR-4537	-0.377	0.003	0.77003717
hsa-miR-1229-3p	-0.26	0.003	0.83508792
hsa-miR-4763-5p	-0.275	0.003	0.82645032
hsa-miR-3978	-0.336	0.003	0.79223481
hsa-miR-3180-3p	-0.389	0.003	0.76365875
hsa-miR-619	-0.377	0.003	0.77003717
hsa-miR-1539	-0.261	0.003	0.83450928
hsa-miRPlus-C1087	-0.249	0.003	0.84147948
hsa-miR-3129-3p	-0.303	0.003	0.81056512
hsa-miR-4281	-0.367	0.003	0.77539321
hsa-miR-548au-3p	-0.267	0.003	0.83104586
hsa-miR-1296	-0.43	0.003	0.74226179
hsa-miR-3186-3p	-0.268	0.003	0.83047002
hsa-miR-4678	-0.404	0.003	0.75575996
hsa-miR-4709-5p	-0.328	0.003	0.7966401
hsa-miR-4647	-0.367	0.003	0.77539321
hsa-miR-4671-3p	-0.347	0.003	0.78621729
hsa-miR-4720-5p	-0.285	0.003	0.82074161
hsa-miR-3917	-0.304	0.003	0.81000347
hsa-miR-4315	-0.342	0.003	0.78894684
hsa-miR-129-2-3p	-0.304	0.003	0.81000347
hsa-miR-3187-3p	-0.412	0.003	0.75158074
ebv-miR-BART17-3p	-0.266	0.003	0.8316221
hsa-miR-4272	-0.308	0.003	0.80776078
hsa-miR-1237-3p	-0.361	0.003	0.77862469
hsa-miR-744-3p	-0.277	0.003	0.82530541
hsa-miR-3928	-0.303	0.003	0.81056512
hsa-miR-30d-3p	-0.291	0.003	0.81733533
ebv-miR-BHRF1-2-5p	-0.269	0.003	0.82989459
hsa-miR-548g-3p	-0.443	0.003	0.73560337

hsa-miR-1265	0.316	0.003	1.24487424
hsa-miR-3913-5p	-0.243	0.003	0.84498638
hsa-miR-1266	-0.378	0.003	0.76950361
hsa-miR-137	-0.31	0.003	0.80664176
hsa-miR-4726-3p	-0.394	0.003	0.76101669
hsa-miR-548al	-0.324	0.003	0.79885192
hsa-miR-5195-5p	-0.347	0.003	0.78621729
hsa-miR-4746-3p	-0.356	0.003	0.78132788
hsa-miR-5579-3p	-0.32	0.003	0.80106988
hsa-miR-4525	-0.302	0.003	0.81112716
hsa-miR-3677-3p	-0.375	0.003	0.77110541
hsa-miR-5092	-0.28	0.003	0.82359102
hsa-miR-558	-0.311	0.003	0.80608283
ebv-miR-BHRF1-3	-0.249	0.003	0.84147948
hsa-miR-3065-3p	-0.311	0.003	0.80608283
hsa-miR-937-3p	-0.301	0.003	0.81168958
hsa-miR-5011-5p	-0.354	0.003	0.78241178
hsa-miR-661	-0.294	0.003	0.81563749
hcmv-miR-US33-5p	-0.338	0.003	0.7911373
hsa-miR-3124-3p	0.739	0.003	1.66901856
hsa-miR-611	-0.342	0.003	0.78894684
hsa-miR-5047	-0.277	0.003	0.82530541
hsa-miR-520g	-0.289	0.003	0.81846918
hsa-miR-545-5p	-0.255	0.003	0.83798713
hsa-miR-4487	-0.303	0.003	0.81056512
hsa-miR-3674	-0.303	0.003	0.81056512
hsa-miR-133a	-0.309	0.003	0.80720107
ebv-miR-BART22	-0.262	0.003	0.83393104
hsa-miR-4638-3p	-0.4	0.003	0.75785828
hsa-miR-4524b-3p	-0.343	0.003	0.78840017
hsa-miR-941	-0.343	0.003	0.78840017
hsa-miR-4740-5p	-0.371	0.003	0.77324634
hsa-miR-5007-5p	-0.283	0.003	0.82188019
hsa-miR-600	0.32	0.003	1.24833055
kshv-miR-K12-10b	-0.327	0.004	0.79719248
hsa-miR-4512	-0.324	0.004	0.79885192
hsa-miR-197-3p	0.306	0.004	1.23627526
hsa-miR-4493	-0.258	0.004	0.8362464
hsa-miR-130a-5p	-0.24	0.004	0.84674531
hsa-miR-4423-5p	0.754	0.004	1.68646222
hsa-miR-4656	-0.316	0.004	0.803294
hsa-miR-1292-5p	-0.336	0.004	0.79223481
hsa-miR-4280	-0.323	0.004	0.79940583
hcmv-miR-UL70-3p	-0.514	0.004	0.70027816
hsa-miR-548at-5p	-0.251	0.004	0.84031375
SNORD38B	1.04	0.004	2.05622765
hsa-miR-1910	-0.269	0.004	0.82989459
hsa-miR-3678-3p	-0.282	0.004	0.82245007
hsa-miR-1911-5p	-0.292	0.004	0.81676899
hsa-miR-519c-3p	-0.317	0.004	0.80273739
hsa-miR-190b	-0.275	0.004	0.82645032
hsa-miR-212-5p	-0.269	0.004	0.82989459
hsa-miR-4690-3p	-0.244	0.004	0.84440089
hsa-miR-330-5p	-0.233	0.004	0.85086373
hsa-miR-1208	-0.352	0.004	0.78349719
hsa-miR-3607-5p	0.663	0.004	1.58337173
hsa-miR-202-3p	-0.411	0.004	0.75210188
hsa-miR-5089-5p	-0.333	0.004	0.79388393

hsa-miR-4761-5p	-0.305	0.004	0.80944222
hsa-miR-4318	-0.242	0.004	0.84557229
hsa-miR-371a-5p	-0.286	0.004	0.82017291
SNORD2	0.674	0.004	1.59549048
hsa-miR-181b-3p	-0.289	0.004	0.81846918
hsa-miR-331-3p	0.334	0.004	1.26050339
hsa-miR-3184-5p	-0.266	0.004	0.8316221
hsa-miR-4727-5p	-0.265	0.004	0.83219873
hsa-miR-3150b-3p	-0.299	0.004	0.8128156
hsa-miR-3615	-0.353	0.004	0.7829543
hsa-miR-129-5p	-0.402	0.004	0.7568084
hsa-miR-3929	-0.241	0.004	0.8461586
hsa-miR-4690-5p	-0.448	0.004	0.73305838
hsa-miR-4693-5p	-0.263	0.004	0.83335321
hsa-miRPlus-J1002	-0.209	0.004	0.86513669
hsa-miR-4775	-0.236	0.004	0.84909625
hsa-miR-4791	0.759	0.004	1.69231719
hsa-miR-2909	-0.306	0.004	0.80888135
hsa-miR-5571-3p	-0.286	0.004	0.82017291
hsa-miR-544a	-0.301	0.004	0.81168958
hsa-miR-3689d	-0.357	0.004	0.78078649
hsa-miR-4689	-0.437	0.004	0.73866903
hsa-miR-423-3p	0.436	0.004	1.35284823
hsa-miR-3649	-0.301	0.004	0.81168958
hsa-miR-4278	-0.253	0.004	0.83914964
hsa-miR-5589-5p	-0.288	0.004	0.8190367
hsa-miR-1301	-0.337	0.004	0.79168587
hsa-miR-1254	-0.204	0.004	0.86814023
hsa-miR-5702	-0.39	0.004	0.7631296
hsa-miR-4741	-0.25	0.004	0.84089642
hsa-miR-3130-3p	-0.31	0.004	0.80664176
hsa-miR-3685	1.421	0.004	2.67771051
hsa-miR-1228-3p	-0.294	0.005	0.81563749
hsa-miR-4665-5p	-0.258	0.005	0.8362464
hsa-miR-4742-5p	-0.392	0.005	0.76207242
hsa-miR-4704-3p	-0.385	0.005	0.765779
hsa-miR-572	-0.359	0.005	0.77970484
hsa-miRPlus-J1003	-0.213	0.005	0.86274134
hsa-miR-4802-5p	-0.356	0.005	0.78132788
hsa-miR-5586-3p	-0.265	0.005	0.83219873
hsa-miR-1343	-0.308	0.005	0.80776078
hsa-miR-1323	-0.282	0.005	0.82245007
kshv-miR-K12-11-3p	-0.235	0.005	0.849685
hsa-miR-646	-0.22	0.005	0.85856544
hsa-miR-5586-5p	-0.286	0.005	0.82017291
hsa-miR-5701	0.582	0.005	1.49692299
hsa-miR-595	-0.345	0.005	0.78730798
hsa-miR-548ah-5p	-0.412	0.005	0.75158074
hsa-miR-4650-5p	-0.303	0.005	0.81056512
SNORD4A	0.607	0.005	1.52308874
hsa-miR-33b-5p	-0.209	0.005	0.86513669
hsa-miR-3129-5p	-0.304	0.005	0.81000347
hsa-miR-634	0.814	0.005	1.75807912
hsa-miR-3650	-0.208	0.005	0.86573657
hsa-miR-1914-5p	-0.279	0.005	0.82416208
hsa-miR-4437	-0.317	0.005	0.80273739
hsa-miR-185-3p	-0.247	0.005	0.84264683
hsa-miR-3135b	-0.289	0.005	0.81846918

hsa-miR-1255b-2-3p	0.585	0.005	1.50003899
hsa-miR-2277-3p	-0.259	0.005	0.83566696
hsa-miR-662	-0.294	0.005	0.81563749
hsa-miR-4676-3p	-0.279	0.005	0.82416208
hsa-miR-5589-3p	-0.373	0.005	0.77217513
hsa-miR-3921	-0.214	0.005	0.86214355
ebv-miR-BART7-3p	-0.356	0.005	0.78132788
hsa-miR-4462	-0.322	0.005	0.79996013
hsa-miR-4740-3p	-0.529	0.005	0.69303494
hsa-miR-3655	-0.274	0.005	0.82702337
hsa-miR-516a-5p	-0.265	0.005	0.83219873
hsa-miR-3156-5p	-0.274	0.005	0.82702337
hsa-miR-520a-3p	-0.309	0.005	0.80720107
hsa-miR-3182	0.784	0.005	1.72189838
hsa-miR-211-5p	-0.303	0.005	0.81056512
hsa-miR-548ao-3p	-0.214	0.005	0.86214355
hcmv-miR-US33-3p	-0.308	0.005	0.80776078
hsa-miR-635	-0.316	0.005	0.803294
hsa-miR-889	-0.328	0.005	0.7966401
hsa-miR-346	-0.263	0.005	0.83335321
hsa-miR-892b	-0.232	0.005	0.85145371
hsa-miR-5590-3p	-0.319	0.005	0.80162533
hsa-miR-637	-0.402	0.005	0.7568084
hsa-miR-5581-5p	-0.485	0.005	0.71449707
hsa-miR-451b	0.242	0.005	1.182631
hsa-miR-4330	-0.192	0.005	0.87539133
kshv-miR-K12-8-3p	-0.261	0.005	0.83450928
hsa-miR-4639-3p	1.084	0.005	2.11990557
hsa-miR-1228-5p	-0.373	0.005	0.77217513
hsa-miR-1204	-0.189	0.005	0.87721355
hsa-miR-615-5p	-0.339	0.005	0.79058912
hsa-miR-4768-5p	0.374	0.005	1.29594097
hsa-miR-1911-3p	-0.334	0.005	0.79333384
SNORD10	0.616	0.005	1.53261996
hsa-miR-4752	-0.32	0.005	0.80106988
hsa-miR-602	-0.328	0.006	0.7966401
hsa-miR-4312	-0.181	0.006	0.88209137
hsa-miR-628-3p	-0.226	0.006	0.85500218
hsa-miR-3648	-0.25	0.006	0.84089642
hsa-miR-3149	0.476	0.006	1.39088197
SNORD3@	0.552	0.006	1.46611676
hsa-miR-3681-5p	-0.277	0.006	0.82530541
hsa-miR-613	-0.336	0.006	0.79223481
RNU5	1.21	0.006	2.31337637
hsa-miR-3605-3p	-0.203	0.006	0.86874219
hsa-miR-371a-3p	-0.293	0.006	0.81620305
hsv2-miR-H2	-0.301	0.006	0.81168958
hsa-miR-4452	-0.307	0.006	0.80832087
hsa-miR-4327	-0.258	0.006	0.8362464
hsa-miR-486-3p	-0.256	0.006	0.83740649
hsa-miR-1225-3p	-0.279	0.006	0.82416208
hsa-miR-4433-5p	-0.33	0.006	0.79553648
hsa-miR-764	-0.236	0.006	0.84909625
hsa-miR-520e	-0.313	0.006	0.80496614
hsa-miR-5584-3p	0.381	0.006	1.30224419
hsa-miR-3684	-0.314	0.006	0.80440837
hsa-miR-4304	-0.244	0.006	0.84440089
hsa-miR-3972	-0.301	0.006	0.81168958

hcmv-miR-US4	-0.281	0.006	0.82302035
hsa-miR-4640-5p	-0.316	0.006	0.803294
ebv-miR-BART18-3p	-0.211	0.006	0.86393819
hsa-miR-154-3p	-0.232	0.006	0.85145371
hsa-miR-200a-5p	-0.206	0.006	0.86693756
hsa-miR-551a	-0.222	0.006	0.85737604
ebv-miR-BART11-5p	-0.337	0.006	0.79168587
hsa-miR-3691-3p	-0.323	0.006	0.79940583
hsa-miR-4275	-0.277	0.006	0.82530541
hsa-miR-3622b-3p	-0.295	0.006	0.81507233
hsa-miR-525-3p	-0.232	0.006	0.85145371
hsa-miR-323b-5p	-0.285	0.006	0.82074161
hsa-miR-1282	-0.285	0.006	0.82074161
hsa-miR-5587-5p	-0.309	0.006	0.80720107
hsa-miR-5687	-0.245	0.006	0.8438158
hsa-miR-4696	-0.319	0.006	0.80162533
hsa-miR-3925-3p	-0.33	0.006	0.79553648
hsa-miR-200b-3p	0.53	0.006	1.4439292
hsa-miR-1255b-5p	-0.256	0.006	0.83740649
hsa-miR-4420	-0.259	0.006	0.83566696
hsa-miR-562	-0.273	0.007	0.82759682
hsa-miR-4650-3p	-0.356	0.007	0.78132788
hsa-let-7f-2-3p	-0.271	0.007	0.8287449
hsa-miR-5685	-0.311	0.007	0.80608283
hsa-miR-4786-3p	-0.247	0.007	0.84264683
hsa-miR-4742-3p	0.419	0.007	1.3370005
hsa-miR-371b-3p	-0.22	0.007	0.85856544
hsa-miR-4676-5p	-0.243	0.007	0.84498638
hsa-miR-1261	-0.369	0.007	0.77431903
hsa-miR-5188	-0.484	0.007	0.71499249
hsa-miR-4498	-0.29	0.007	0.81790206
hsa-miR-1825	-0.311	0.007	0.80608283
hsa-miR-1238-3p	-0.266	0.007	0.8316221
hsa-miR-671-5p	-0.425	0.007	0.74483873
hsa-miR-383	-0.263	0.007	0.83335321
RNU1	0.94	0.007	1.91852824
hsa-miR-891a	-0.335	0.007	0.79278414
hsv2-miR-H23-5p	-0.246	0.007	0.84323111
hsa-miR-543	-0.25	0.007	0.84089642
hsa-miR-4681	-0.291	0.007	0.81733533
hsa-miR-588	-0.228	0.007	0.85381771
hsa-miR-1200	-0.231	0.007	0.8520441
hsa-miR-3941	0.787	0.007	1.72548269
hsa-miR-509-3-5p	-0.276	0.007	0.82587766
hsa-miR-549a	-0.436	0.007	0.73918122
hsa-miR-548c-3p	-0.279	0.007	0.82416208
hsa-miR-4781-3p	-0.322	0.007	0.79996013
hsa-miR-1207-3p	-0.255	0.007	0.83798713
hsa-miR-34a-3p	-0.189	0.007	0.87721355
hcmv-miR-UL36-3p	-0.242	0.007	0.84557229
hsa-miR-5006-3p	0.38	0.007	1.30134186
hsa-miR-539-3p	-0.355	0.007	0.78186964
kshv-miR-K12-4-3p	-0.188	0.007	0.8778218
hsa-miR-326	-0.237	0.007	0.8485079
hcmv-miR-US5-2	-0.339	0.007	0.79058912
kshv-miR-K12-9-5p	-0.222	0.007	0.85737604
ebv-miR-BART19-5p	-0.22	0.007	0.85856544
hsa-miR-3124-5p	-0.48	0.007	0.71697762

hcmv-miR-UL36-5p	-0.262	0.007	0.83393104
hsa-miR-3121-5p	-0.275	0.007	0.82645032
hsa-miR-3909	-0.252	0.007	0.83973149
hsa-miR-621	-0.271	0.007	0.8287449
hsa-miR-924	-0.342	0.007	0.78894684
hsa-miR-300	-0.308	0.007	0.80776078
hsa-miR-4732-5p	-0.602	0.007	0.65883998
ebv-miR-BART4-3p	-0.246	0.007	0.84323111
hsa-miR-362-5p	-0.335	0.007	0.79278414
hsa-miR-4668-3p	-0.26	0.007	0.83508792
hsa-miR-4728-3p	0.304	0.007	1.23456261
hsa-miR-548x-3p	-0.366	0.007	0.77593085
hsa-miR-4528	-0.25	0.007	0.84089642
hsa-miR-3680-5p	1.148	0.007	2.2160647
hsa-miR-642b-5p	0.652	0.007	1.57134503
hsa-miR-3148	0.527	0.007	1.44092975
SNORD13	0.934	0.007	1.91056587
hsa-miR-3916	-0.222	0.007	0.85737604
hsa-miR-1471	-0.249	0.007	0.84147948
hsa-miR-5581-3p	0.224	0.007	1.16796739
hsa-miR-19a-5p	-0.273	0.007	0.82759682
hsa-miR-653	-0.268	0.008	0.83047002
hsa-miR-4273	-0.25	0.008	0.84089642
hsa-miR-3937	-0.316	0.008	0.803294
SNORD48	0.814	0.008	1.75807912
hsa-miR-524-3p	-0.243	0.008	0.84498638
hiv1-miR-H1	-0.335	0.008	0.79278414
hsa-miR-940	-0.216	0.008	0.86094919
hsa-miR-4302	-0.24	0.008	0.84674531
hsa-miR-3591-5p	0.389	0.008	1.30948542
hsv1-miR-H3-5p	-0.17	0.008	0.88884268
hsa-miR-4667-5p	0.652	0.008	1.57134503
hsa-miR-302d-3p	-0.231	0.008	0.8520441
hsa-miR-670	-0.26	0.008	0.83508792
hsa-miR-4722-3p	-0.248	0.008	0.84206295
SNORD44	1.149	0.008	2.21760129
hsv1-miR-H4-3p	0.236	0.008	1.17772279
hsa-miR-520c-3p	-0.252	0.008	0.83973149
hsa-miR-515-5p	-0.277	0.008	0.82530541
hsa-miR-523-3p	-0.229	0.008	0.8532261
hsa-miR-4670-3p	-0.219	0.008	0.85916075
hsa-miR-4637	-0.221	0.008	0.85797053
hsa-miR-200c-3p	0.541	0.008	1.45498068
hsa-miR-148b-5p	-0.251	0.008	0.84031375
hsa-miR-3176	-0.275	0.008	0.82645032
hsa-miR-571	-0.224	0.008	0.85618828
hsa-miR-4730	-0.447	0.008	0.73356667
hsa-miR-4783-5p	-0.31	0.008	0.80664176
hsa-miR-579	-0.309	0.008	0.80720107
hsa-miR-1233-3p	-0.239	0.008	0.84733243
hsa-miR-4500	0.725	0.008	1.65290064
hsa-miR-4519	-0.172	0.008	0.88761134
hsa-miR-548ab	-0.256	0.008	0.83740649
hsa-miR-4632-3p	-0.255	0.008	0.83798713
hsa-miR-4799-5p	-0.341	0.008	0.78949389
hsa-miR-2355-5p	-0.296	0.008	0.81450756
kshv-miR-K12-7-5p	-0.276	0.008	0.82587766
hsa-miR-188-3p	-0.353	0.008	0.7829543

hsa-miR-524-5p	-0.309	0.008	0.80720107
hsa-miR-4269	-0.22	0.008	0.85856544
hsa-miR-4308	-0.156	0.008	0.89751005
hsa-miR-3199	-0.319	0.008	0.80162533
hsa-miR-339-3p	-0.349	0.008	0.78512812
hsa-miR-4684-5p	-0.268	0.008	0.83047002
hsa-miR-554	-0.182	0.008	0.88148016
hsa-miR-331-5p	-0.361	0.008	0.77862469
hsa-miR-522-3p	-0.223	0.008	0.85678195
hsa-miR-1294	-0.233	0.008	0.85086373
hsa-miR-2682-5p	-0.226	0.008	0.85500218
hsa-miR-511	-0.215	0.008	0.86154616
hsa-miR-5708	-0.417	0.008	0.74898047
hsa-miR-4536-3p	-0.278	0.008	0.82473355
hsa-miR-4539	-0.299	0.008	0.8128156
hsa-miR-4760-5p	-0.237	0.008	0.8485079
hsa-miR-1182	-0.329	0.008	0.7960881
hsa-miR-3193	-0.193	0.008	0.87478476
hsa-miR-4699-3p	-0.238	0.008	0.84791996
SNORD110	0.613	0.008	1.52943628
hsa-miR-3191-3p	-0.276	0.008	0.82587766
hsa-miR-548b-3p	-0.339	0.008	0.79058912
hsa-miR-33b-3p	-0.232	0.008	0.85145371
hsa-miR-4466	-0.289	0.009	0.81846918
sv40-miR-S1-3p	-0.316	0.009	0.803294
hsa-miR-3919	-0.311	0.009	0.80608283
hsa-miR-767-3p	-0.329	0.009	0.7960881
hsa-miR-490-5p	-0.267	0.009	0.83104586
hsa-miR-4727-3p	-0.317	0.009	0.80273739
hsa-miR-5186	-0.314	0.009	0.80440837
hsa-miR-514a-5p	-0.216	0.009	0.86094919
hcmv-miR-UL22A-5p	-0.389	0.009	0.76365875
hsa-miR-4422	-0.346	0.009	0.78676245
hsa-miR-541-3p	-0.309	0.009	0.80720107
hsa-miR-561-5p	-0.242	0.009	0.84557229
hsa-miR-182-3p	-0.301	0.009	0.81168958
hsa-miR-574-5p	0.55	0.009	1.4640857
hsa-miR-526b-3p	-0.196	0.009	0.87296759
hsa-miR-199b-5p	0.986	0.009	1.98068574
hsa-miRPlus-D1058	-0.324	0.009	0.79885192
hsa-miR-548as-5p	-0.257	0.009	0.83682624
hsa-miR-3140-3p	-0.236	0.009	0.84909625
hsa-miR-4524a-5p	-0.261	0.009	0.83450928
hsa-miR-3167	-0.237	0.009	0.8485079
hsa-miR-219-5p	-0.263	0.009	0.83335321
hsa-miR-4520b-3p	-0.372	0.009	0.77271055
hsa-miR-4711-5p	-0.324	0.009	0.79885192
hcmv-miR-UL22A-3p	-0.247	0.009	0.84264683
hsa-miR-4482-5p	-0.25	0.009	0.84089642
hsa-miR-370	-0.279	0.009	0.82416208
hsa-miR-5579-5p	-0.236	0.009	0.84909625
hsa-miR-2861	-0.258	0.009	0.8362464
hsa-miR-4686	-0.265	0.009	0.83219873
hsa-miR-218-2-3p	-0.277	0.009	0.82530541
hsa-miR-3177-3p	-0.223	0.009	0.85678195
hsa-miR-4469	-0.252	0.009	0.83973149
hsa-miR-4662a-5p	-0.245	0.009	0.8438158
hsa-miR-4471	-0.295	0.009	0.81507233

hsa-miR-2116-3p	-0.171	0.009	0.8882268
hsa-miR-603	-0.332	0.009	0.7944344
hsa-miR-187-5p	-0.392	0.009	0.76207242
hsa-miR-4440	-0.31	0.009	0.80664176
hsa-miR-649	-0.236	0.009	0.84909625
hsv2-miR-H19	-0.177	0.009	0.88454044
hsa-miR-183-5p	0.202	0.009	1.15029189
hsa-miR-2277-5p	-0.299	0.009	0.8128156
hsa-miR-448	-0.238	0.009	0.84791996
hsa-miR-640	-0.23	0.009	0.85263489
hsa-miR-3170	-0.232	0.009	0.85145371
hsa-miR-4697-3p	-0.319	0.009	0.80162533
hsa-let-7i-3p	-0.215	0.009	0.86154616
hsa-miR-1537	-0.271	0.009	0.8287449
hsv2-miR-H6-3p	0.403	0.009	1.32225461
hsa-miR-564	-0.313	0.009	0.80496614
hsa-miR-769-5p	-0.184	0.01	0.88025901
hsa-miR-3939	-0.249	0.01	0.84147948
hsa-miR-1185-1-3p	-0.285	0.01	0.82074161
hsa-miR-5700	-0.268	0.01	0.83047002
hsa-miR-639	-0.261	0.01	0.83450928
hsa-miR-1205	-0.374	0.01	0.77164009
hsa-miR-3667-5p	1.203	0.01	2.30217898
kshv-miR-K12-12-5p	-0.281	0.01	0.82302035
hsa-miR-5008-3p	-0.414	0.01	0.75053955
hsa-miR-378c	-0.231	0.01	0.8520441
hsa-miR-1306-3p	-0.23	0.01	0.85263489
hsa-miR-3713	-0.345	0.01	0.78730798
hsa-miR-642a-5p	-0.215	0.01	0.86154616
hsa-miR-5582-3p	-0.247	0.01	0.84264683
hsa-miR-4691-5p	-0.377	0.01	0.77003717
hsa-miR-15b-3p	-0.26	0.01	0.83508792
jcv-miR-J1-5p	-0.277	0.01	0.82530541
hsa-miR-3907	-0.248	0.01	0.84206295
hsa-miR-4759	-0.267	0.01	0.83104586
hsa-miR-365b-5p	-0.188	0.01	0.8778218
hsa-miR-4733-5p	-0.337	0.01	0.79168587
hcmv-miR-UL70-5p	-0.235	0.01	0.849685
ebv-miR-BART3-5p	-0.293	0.01	0.81620305
hsa-miR-4268	0.709	0.01	1.63467066
hsa-miR-4787-3p	-0.188	0.01	0.8778218
hsa-miR-1305	-0.243	0.01	0.84498638
hsa-miR-4263	-0.253	0.01	0.83914964
hsa-miR-5583-3p	-0.224	0.01	0.85618828
hsa-miR-30b-5p	0.896	0.01	1.86089932
hsa-miR-4659b-5p	-0.205	0.01	0.86753869
hsa-miR-548j	-0.28	0.01	0.82359102
RNU6-1_RNU6-2	0.391	0.01	1.31130201
hsa-miR-4697-5p	-0.244	0.01	0.84440089
hsa-miR-23c	0.811	0.01	1.7544271
hsa-miR-660-5p	0.391	0.01	1.31130201
hsa-miR-548a-5p	-0.21	0.01	0.86453723
hsa-miR-875-3p	-0.153	0.011	0.89937831
hsa-miR-548ak	-0.295	0.011	0.81507233
hsa-miR-3074-3p	-0.375	0.011	0.77110541
hsa-miR-4283	-0.347	0.011	0.78621729
hsa-miR-3616-3p	-0.226	0.011	0.85500218
hsa-miR-4295	-0.244	0.011	0.84440089

hsa-miR-4431	-0.302	0.011	0.81112716
kshv-miR-K12-3-3p	-0.244	0.011	0.84440089
hsa-miR-1322	-0.343	0.011	0.78840017
hsa-miR-4538	-0.245	0.011	0.8438158
hsa-miR-4762-3p	-0.313	0.011	0.80496614
hsa-miR-15b-5p	0.691	0.011	1.61440215
hsa-miR-4743-5p	-0.197	0.011	0.87236271
hsa-miR-548aq-5p	-0.247	0.011	0.84264683
hsa-miR-3186-5p	-0.262	0.011	0.83393104
hsa-miR-563	-0.259	0.011	0.83566696
hsa-miR-196a-3p	0.285	0.011	1.21841026
hsa-miR-939-5p	-0.277	0.011	0.82530541
hsa-miR-548o-3p	-0.175	0.011	0.88576752
hsa-miR-378h	-0.231	0.011	0.8520441
hsa-miR-4455	0.555	0.011	1.46916863
hsa-miR-548ah-3p	-0.252	0.011	0.83973149
hsa-miRPlus-G1065-5p	-0.231	0.011	0.8520441
hsa-miR-200a-3p	0.7	0.011	1.62450479
hsa-miR-651	-0.393	0.011	0.76154437
hsa-miR-2053	-0.26	0.011	0.83508792
hsa-miR-4446-5p	-0.306	0.011	0.80888135
hsa-miR-4766-3p	-0.231	0.011	0.8520441
hsa-miR-483-3p	0.472	0.011	1.38703097
hsa-miR-4661-5p	-0.17	0.011	0.88884268
hsa-miR-103b	-0.168	0.011	0.89007573
hsa-miR-128	-0.17	0.011	0.88884268
hsa-miR-1538	-0.195	0.011	0.8735729
hsa-miR-4486	-0.291	0.011	0.81733533
hsa-miR-1293	-0.215	0.011	0.86154616
hsa-miR-10a-5p	0.911	0.011	1.88034841
hsa-miR-4290	0.626	0.011	1.54328018
hsa-miR-1251	-0.309	0.011	0.80720107
hsa-miR-433	-0.241	0.011	0.8461586
hsa-miR-1468	-0.277	0.011	0.82530541
hsa-miR-570-3p	-0.239	0.011	0.84733243
hsa-miR-519b-3p	-0.182	0.011	0.88148016
kshv-miR-K12-10a-3p	-0.278	0.011	0.82473355
hsv2-miR-H23-3p	-0.248	0.011	0.84206295
hsa-miR-584-3p	-0.296	0.011	0.81450756
hsa-miR-568	-0.228	0.011	0.85381771
kshv-miR-K12-9-3p	-0.181	0.011	0.88209137
hsa-miR-5583-5p	-0.271	0.011	0.8287449
hsa-miR-4642	-0.232	0.011	0.85145371
hsa-miR-1245b-3p	-0.21	0.011	0.86453723
hsa-miR-4771	-0.232	0.011	0.85145371
hsa-miR-3977	-0.292	0.011	0.81676899
hsa-miR-195-3p	-0.289	0.011	0.81846918
hsa-miR-4786-5p	-0.308	0.011	0.80776078
hsa-miR-198	-0.217	0.011	0.86035263
hsa-miR-3156-3p	1.199	0.012	2.29580483
hsa-miR-3675-5p	-0.262	0.012	0.83393104
hsa-miR-4325	-0.155	0.012	0.89813237
hsa-miR-517c-3p	-0.208	0.012	0.86573657
hsa-miR-548e	-0.189	0.012	0.87721355
hsa-miR-671-3p	-0.272	0.012	0.82817066
hsa-miRPlus-A1086	0.921	0.012	1.89342726
hsa-miR-4670-5p	-0.22	0.012	0.85856544

hsa-miR-3153	-0.328	0.012	0.7966401
hsa-miR-330-3p	-0.235	0.012	0.849685
hsa-miR-548b-5p	-0.239	0.012	0.84733243
hsa-miR-4798-3p	-0.3	0.012	0.8122524
hsa-miR-4703-3p	-0.23	0.012	0.85263489
hsa-miR-557	-0.179	0.012	0.88331505
hsa-miR-660-3p	0.697	0.012	1.62113024
hsa-miRPlus-J1005	-0.2	0.012	0.87055056
hsa-miR-3664-5p	0.191	0.012	1.14155471
hsa-miR-450a-5p	-0.19	0.012	0.87660572
ebv-miR-BART21-3p	-0.222	0.012	0.85737604
hsa-miR-363-5p	0.381	0.012	1.30224419
hsa-miR-412	-0.163	0.012	0.89316585
hsa-miR-548ax	-0.249	0.012	0.84147948
hsa-miR-1295b-3p	-0.264	0.012	0.83277577
hsa-miR-3194-5p	-0.246	0.012	0.84323111
hsa-miR-526b-5p	-0.243	0.013	0.84498638
hsa-miRPlus-A1031	-0.264	0.013	0.83277577
hsa-miR-550b-2-5p	-0.242	0.013	0.84557229
hsa-miR-4313	-0.233	0.013	0.85086373
hsa-miR-296-5p	-0.18	0.013	0.882703
hsa-miR-5009-5p	-0.294	0.013	0.81563749
hsa-miR-4692	-0.289	0.013	0.81846918
hsa-miR-4254	-0.232	0.013	0.85145371
hsa-miR-5693	-0.244	0.013	0.84440089
hsa-miR-615-3p	-0.173	0.013	0.88699631
hsa-miR-4442	-0.253	0.013	0.83914964
hsa-miR-4671-5p	-0.178	0.013	0.88392753
hsa-miR-210	0.262	0.013	1.19913991
hsa-miR-5008-5p	-0.294	0.013	0.81563749
hsa-miR-5704	1.08	0.013	2.11403608
hsa-miR-3677-5p	-0.292	0.013	0.81676899
hsa-miR-548ao-5p	-0.45	0.013	0.73204285
ebv-miR-BART12	-0.183	0.013	0.88086937
hsa-miR-362-3p	0.309	0.013	1.2388487
hsa-miR-888-3p	-0.211	0.013	0.86393819
hsa-miR-935	-0.201	0.013	0.86994735
hsa-miR-4499	-0.242	0.013	0.84557229
hsa-miR-3659	-0.256	0.013	0.83740649
hsa-miR-4738-3p	-0.273	0.013	0.82759682
hsa-miR-3131	-0.197	0.013	0.87236271
hsa-miR-548m	-0.263	0.013	0.83335321
ebv-miR-BART20-3p	-0.278	0.013	0.82473355
hsa-miR-5003-3p	-0.193	0.013	0.87478476
hsa-miR-518d-3p	-0.314	0.013	0.80440837
hsa-miR-5580-3p	-0.227	0.013	0.85440974
hsa-miR-1269a	-0.281	0.014	0.82302035
hsa-miR-655	-0.194	0.014	0.87417862
hsa-miR-548l	-0.16	0.014	0.89502507
ebv-miR-BART2-5p	-0.18	0.014	0.882703
hsa-miR-610	-0.221	0.014	0.85797053
hsa-miR-3120-5p	-0.213	0.014	0.86274134
hsa-miR-3189-5p	-0.177	0.014	0.88454044
hsa-miR-1324	-0.188	0.014	0.8778218
hsa-miR-514b-3p	-0.27	0.014	0.82931955
hsa-miR-4259	-0.196	0.014	0.87296759
hsa-miR-4713-3p	-0.187	0.014	0.87843047
hsa-miR-3610	-0.238	0.014	0.84791996

hsa-miR-1185-5p	-0.171	0.014	0.8882268
hsa-miR-4461	-0.257	0.014	0.83682624
hsa-miRPlus-J1004	-0.199	0.014	0.87115419
hsa-miR-4677-5p	-0.291	0.014	0.81733533
hsa-miR-663b	-0.28	0.014	0.82359102
hsa-miR-4758-5p	-0.425	0.014	0.74483873
hsa-miR-500a-3p	0.246	0.014	1.1859145
hsa-let-7f-5p	0.558	0.014	1.47222686
hsa-miR-4492	-0.266	0.014	0.8316221
hsa-miR-3922-5p	-0.245	0.014	0.8438158
hsa-miR-29b-3p	0.585	0.014	1.50003899
hsa-miR-5682	-0.297	0.014	0.81394319
hsa-miR-1183	-0.272	0.014	0.82817066
hsa-miR-3664-3p	-0.322	0.014	0.79996013
hsa-miR-2276	-0.274	0.014	0.82702337
hsa-miR-130b-5p	0.249	0.014	1.18838311
hsa-miR-4782-3p	-0.22	0.015	0.85856544
hsa-miR-4796-5p	-0.222	0.015	0.85737604
hsa-miR-1206	-0.356	0.015	0.78132788
hsa-miR-4284	1.195	0.015	2.28944832
hsa-miR-491-3p	0.371	0.015	1.29324893
hsa-miR-4780	0.71	0.015	1.63580412
hsa-miR-4310	-0.215	0.015	0.86154616
hsa-miR-548y	-0.25	0.015	0.84089642
hsa-miR-550a-5p	-0.274	0.015	0.82702337
hsa-miR-4712-5p	-0.352	0.015	0.78349719
hsa-miR-4319	-0.202	0.015	0.86934456
hsa-miR-3197	-0.279	0.015	0.82416208
hsa-miR-378g	-0.262	0.015	0.83393104
hsa-miR-124-5p	-0.194	0.015	0.87417862
hsa-miR-4473	0.351	0.015	1.27544439
SNORD6	0.491	0.015	1.4054187
hsa-miR-4745-3p	-0.271	0.015	0.8287449
ebv-miR-BART2-3p	-0.335	0.015	0.79278414
hsa-miR-4634	-0.287	0.015	0.81960461
hsa-miR-624-5p	-0.172	0.015	0.88761134
hsa-miR-5686	-0.192	0.015	0.87539133
hsa-miR-4774-3p	-0.264	0.015	0.83277577
kshv-miR-K12-1-5p	-0.181	0.015	0.88209137
hsa-miR-224-5p	-0.191	0.015	0.87599832
hsa-miR-4435	-0.209	0.015	0.86513669
hsa-miR-181d	0.388	0.015	1.30857807
hsa-miR-367-3p	-0.322	0.015	0.79996013
hsa-miR-3200-5p	-0.219	0.015	0.85916075
hsa-miR-96-3p	-0.23	0.015	0.85263489
hsa-miR-212-3p	-0.184	0.015	0.88025901
hsa-miR-30c-2-3p	-0.216	0.015	0.86094919
hsa-miR-1470	-0.205	0.015	0.86753869
hsa-miR-3652	-0.24	0.015	0.84674531
hsa-miR-106a-5p	0.539	0.015	1.45296505
hsa-miR-4680-3p	-0.16	0.015	0.89502507
hsv2-miR-H22	-0.263	0.015	0.83335321
hsa-miR-584-5p	-0.148	0.015	0.90250073
hsa-miR-4707-3p	-0.214	0.015	0.86214355
ebv-miR-BART4-5p	-0.244	0.015	0.84440089
hsv2-miR-H3	-0.219	0.016	0.85916075
hsa-miR-3147	-0.286	0.016	0.82017291
hsa-miR-4688	-0.222	0.016	0.85737604

hsa-miR-181a-5p	0.417	0.016	1.3351483
hsa-miR-4477b	-0.289	0.016	0.81846918
hsa-miR-1255a	0.583	0.016	1.49796093
hsa-miR-3692-5p	-0.301	0.016	0.81168958
hsa-miR-506-5p	-0.261	0.016	0.83450928
hsa-miR-556-5p	-0.192	0.016	0.87539133
hsa-miR-4501	-0.391	0.016	0.76260083
hsv1-miR-H8-3p	-0.156	0.016	0.89751005
hsa-miR-507	-0.191	0.016	0.87599832
hsa-miR-936	-0.258	0.016	0.8362464
hsa-miR-4763-3p	-0.233	0.016	0.85086373
hsa-miR-361-3p	-0.234	0.016	0.85027416
hsa-miR-4655-3p	-0.275	0.016	0.82645032
hsa-miR-555	-0.196	0.016	0.87296759
hsa-miR-4754	-0.188	0.016	0.8778218
hsa-miR-1914-3p	-0.24	0.016	0.84674531
hsa-miR-138-5p	-0.167	0.016	0.8906929
hsa-miR-5680	-0.209	0.016	0.86513669
ebv-miR-BART7-5p	-0.233	0.016	0.85086373
hsa-miR-624-3p	-0.217	0.016	0.86035263
hsa-miR-544b	-0.259	0.016	0.83566696
hsa-miR-4639-5p	-0.199	0.016	0.87115419
hsa-miR-5587-3p	-0.201	0.016	0.86994735
hsa-miR-150-5p	0.32	0.016	1.24833055
hsa-miR-4768-3p	-0.349	0.016	0.78512812
hsa-miR-24-3p	0.84	0.016	1.79005014
hsa-miR-30a-5p	0.502	0.016	1.41617544
hsa-miR-4460	-0.257	0.016	0.83682624
hsa-miR-876-5p	-0.223	0.017	0.85678195
hsa-miR-4320	-0.185	0.017	0.87964908
hsa-miR-4489	-0.314	0.017	0.80440837
hsa-miR-873-5p	-0.196	0.017	0.87296759
hsa-miR-488-3p	-0.228	0.017	0.85381771
hsa-let-7d-5p	0.607	0.017	1.52308874
hsa-miR-5591-3p	-0.158	0.017	0.8962667
hsa-miR-4745-5p	-0.279	0.017	0.82416208
hsa-miR-432-5p	-0.348	0.017	0.78567252
hsa-miR-4673	-0.316	0.017	0.803294
ebv-miR-BART16	0.349	0.017	1.27367748
hsa-miR-548n	-0.248	0.017	0.84206295
hsa-miR-3192	-0.309	0.017	0.80720107
hsa-miR-4264	-0.185	0.017	0.87964908
hsa-miR-718	-0.195	0.017	0.8735729
hsa-miR-520d-5p	-0.321	0.017	0.80051481
hsa-miR-4718	-0.251	0.017	0.84031375
hsa-miR-1245a	-0.306	0.017	0.80888135
hsa-miR-4684-3p	-0.245	0.017	0.8438158
hsa-miR-4506	-0.275	0.017	0.82645032
hsa-miR-3115	-0.208	0.017	0.86573657
hsa-miR-3944-5p	-0.224	0.017	0.85618828
hsa-miR-489	-0.131	0.018	0.91319825
hsa-miR-1263	-0.176	0.018	0.88515376
hsa-miR-3158-3p	-0.223	0.018	0.85678195
ebv-miR-BART10-5p	-0.21	0.018	0.86453723
hsa-miR-5694	-0.193	0.018	0.87478476
hsa-miR-129-1-3p	0.245	0.018	1.18509277
hsa-miR-650	-0.189	0.018	0.87721355
hsa-miR-1181	-0.232	0.018	0.85145371

hsa-miR-148a-5p	-0.172	0.018	0.88761134
hsa-miR-3607-3p	0.518	0.018	1.43196874
hsa-miR-4276	-0.183	0.018	0.88086937
hsa-miR-4789-3p	-0.23	0.018	0.85263489
hsa-miR-3189-3p	-0.144	0.018	0.90500646
ebv-miR-BART9-3p	-0.313	0.018	0.80496614
hsa-miR-4665-3p	-0.197	0.018	0.87236271
hsa-miR-4494	-0.227	0.018	0.85440974
hsa-miR-2467-3p	-0.249	0.018	0.84147948
hsa-miR-411-3p	-0.211	0.018	0.86393819
hsa-miR-4450	0.247	0.018	1.1867368
hsa-miR-23b-3p	1.068	0.018	2.09652495
hsa-miR-1972	-0.211	0.018	0.86393819
hsa-miR-325	-0.233	0.019	0.85086373
hsa-miR-514a-3p	-0.178	0.019	0.88392753
hsa-miR-539-5p	-0.199	0.019	0.87115419
hsa-miR-302a-5p	-0.342	0.019	0.78894684
hsa-miR-4427	-0.194	0.019	0.87417862
hsv2-miR-H9-5p	-0.199	0.019	0.87115419
hsa-miR-1295a	-0.164	0.019	0.89254697
hsa-miR-4517	-0.222	0.019	0.85737604
hsa-miR-3689a-3p	-0.233	0.019	0.85086373
hsa-miR-636	-0.191	0.019	0.87599832
hsa-miR-19b-2-5p	-0.181	0.019	0.88209137
hsa-miR-23a-3p	0.634	0.019	1.55186171
hsa-miR-4703-5p	-0.268	0.019	0.83047002
hsa-miR-596	-0.221	0.019	0.85797053
hsa-miR-141-3p	0.534	0.019	1.44793817
hsa-miR-643	-0.277	0.019	0.82530541
hsa-miR-607	-0.225	0.019	0.85559503
hsv1-miR-H3-3p	-0.19	0.019	0.87660572
hsa-miR-4749-3p	-0.188	0.019	0.8778218
hsa-miR-367-5p	-0.213	0.019	0.86274134
hsa-miR-342-3p	0.566	0.019	1.4804133
hsa-miR-3942-3p	-0.247	0.019	0.84264683
hsa-miR-4753-5p	-0.311	0.019	0.80608283
hsa-miR-592	-0.217	0.019	0.86035263
hsa-miR-19a-3p	0.476	0.019	1.39088197
hsa-miR-3666	-0.244	0.019	0.84440089
hsa-miRPlus-K1303*	-0.193	0.019	0.87478476
hsa-miR-4641	-0.222	0.019	0.85737604
hsa-miR-499b-5p	-0.198	0.019	0.87175824
hsa-miR-105-5p	-0.168	0.019	0.89007573
hsa-miR-1245b-5p	-0.328	0.019	0.7966401
hsa-miR-548s	-0.174	0.019	0.8863817
hsa-miR-499a-5p	-0.247	0.02	0.84264683
hsa-miR-922	-0.142	0.02	0.90626194
hsa-miR-618	-0.181	0.02	0.88209137
hsa-miR-4326	-0.186	0.02	0.87903956
hsa-miR-515-3p	-0.3	0.02	0.8122524
hsa-miR-4482-3p	-0.302	0.02	0.81112716
hsa-miR-518f-5p	-0.183	0.02	0.88086937
hsa-miR-4257	-0.224	0.02	0.85618828
hsa-miR-452-3p	-0.252	0.02	0.83973149
hsa-miR-5681b	0.574	0.02	1.48864526
SNORD68	0.584	0.02	1.4989996
hsa-miR-642a-3p	-0.325	0.02	0.79829839
hsa-miR-338-5p	-0.197	0.02	0.87236271

hsa-miR-4776-5p	-0.238	0.02	0.84791996
hsa-miR-503-3p	-0.174	0.02	0.8863817
hsa-miR-4731-3p	-0.288	0.02	0.8190367
hsa-miR-4705	-0.172	0.02	0.88761134
hsa-miR-4680-5p	-0.229	0.02	0.8532261
hsa-miR-297	-0.156	0.02	0.89751005
hsa-miR-508-3p	-0.159	0.02	0.89564567
hsa-miR-4418	-0.282	0.02	0.82245007
hsa-miR-30d-5p	0.463	0.02	1.37840515
hsa-miR-107	0.348	0.02	1.27279494
hsa-miR-487a	-0.234	0.02	0.85027416
hsa-miR-3169	-0.216	0.02	0.86094919
hsa-miR-4737	-0.274	0.02	0.82702337
hsa-miR-3064-5p	-0.201	0.02	0.86994735
hsa-miR-4534	-0.392	0.02	0.76207242
hsa-miR-3613-5p	-0.138	0.02	0.90877812
hsa-miR-4292	-0.215	0.02	0.86154616
hsa-miR-30c-5p	0.756	0.021	1.68880177
hsa-let-7a-2-3p	-0.169	0.021	0.88945899
hsv2-miR-H21	-0.189	0.021	0.87721355
hsa-miR-191-3p	-0.164	0.021	0.89254697
hsa-miR-518f-3p	-0.221	0.021	0.85797053
hsa-miR-1224-5p	-0.258	0.021	0.8362464
hsa-miR-1267	-0.216	0.021	0.86094919
ebv-miR-BART5-5p	-0.216	0.021	0.86094919
hsa-miR-1285-5p	0.296	0.021	1.22773568
hsa-miR-4772-3p	-0.259	0.021	0.83566696
hsa-miR-103a-2-5p	-0.136	0.021	0.91003882
hsa-miR-3200-3p	-0.279	0.021	0.82416208
hsa-miR-1271-3p	-0.31	0.021	0.80664176
hsa-miR-3127-5p	-0.292	0.021	0.81676899
hsa-miR-5195-3p	-0.172	0.021	0.88761134
hsa-miR-4643	-0.231	0.021	0.8520441
hsa-miR-1299	0.259	0.021	1.19664896
hsa-miR-223-5p	-0.182	0.021	0.88148016
hsv2-miR-H5	-0.185	0.021	0.87964908
hsa-miR-4672	-0.173	0.021	0.88699631
hsa-miR-146b-3p	-0.185	0.021	0.87964908
hsa-miR-373-5p	-0.134	0.021	0.91130128
hsa-miR-3136-5p	-0.211	0.022	0.86393819
hsa-miR-219-2-3p	-0.612	0.022	0.65428904
hsa-miR-31-5p	0.287	0.022	1.22010051
hsa-miR-3663-3p	-0.205	0.022	0.86753869
hsa-miR-379-3p	-0.296	0.022	0.81450756
hsa-miR-599	-0.209	0.022	0.86513669
SNORD15A	-0.309	0.022	0.80720107
hsa-miR-676-3p	-0.158	0.022	0.8962667
hsa-miR-520a-5p	-0.226	0.022	0.85500218
hsa-miR-338-3p	-0.138	0.022	0.90877812
hsa-miR-762	-0.229	0.022	0.8532261
hsa-miR-302b-5p	-0.154	0.022	0.89875513
hsa-miR-340-3p	0.207	0.022	1.15428542
hsa-miR-3074-5p	-0.173	0.023	0.88699631
hsa-miR-3138	-0.254	0.023	0.83856818
hsa-miR-4715-3p	-0.217	0.023	0.86035263
hsa-miR-3136-3p	1.08	0.023	2.11403608
hsa-miR-548ar-3p	-0.181	0.023	0.88209137
hsa-miR-4457	-0.256	0.023	0.83740649

hsa-miR-3908	-0.219	0.023	0.85916075
hsa-miR-4480	-0.33	0.024	0.79553648
hsa-miR-499b-3p	-0.216	0.024	0.86094919
hsa-miR-3665	-0.304	0.024	0.81000347
hsa-miR-3185	-0.234	0.024	0.85027416
hsa-miR-4522	-0.319	0.024	0.80162533
hsa-miR-3618	-0.204	0.024	0.86814023
hsa-miR-18a-3p	-0.17	0.024	0.88884268
hsa-miR-548ag	-0.293	0.024	0.81620305
kshv-miR-K12-4-5p	-0.151	0.024	0.90062598
hsa-miR-1253	-0.171	0.024	0.8882268
RNU6-1	0.363	0.024	1.28609748
hsa-miR-5009-3p	-0.173	0.024	0.88699631
SNORD49A	0.592	0.024	1.50733491
hsa-miR-548w	-0.209	0.025	0.86513669
hsa-miR-3663-5p	-0.218	0.025	0.85975649
hsa-miR-890	-0.238	0.025	0.84791996
hsv2-miR-H13	-0.231	0.025	0.8520441
hsa-miR-3139	-0.131	0.025	0.91319825
hsa-miR-877-3p	0.232	0.025	1.17446197
hsa-miR-1203	-0.248	0.025	0.84206295
hsa-miR-4287	-0.155	0.025	0.89813237
hcmv-miR-US25-2-5p	-0.282	0.025	0.82245007
hsa-miR-3188	-0.176	0.025	0.88515376
hsa-miR-497-5p	0.461	0.025	1.3764956
hsa-miR-30e-5p	0.681	0.025	1.60325066
kshv-miR-K12-2-5p	-0.242	0.025	0.84557229
hsa-miR-4766-5p	-0.235	0.025	0.849685
hsa-miR-222-5p	-0.181	0.025	0.88209137
hsa-miR-4291	0.333	0.025	1.25962998
hsv2-miR-H11-5p	-0.162	0.025	0.89378516
kshv-miR-K12-8-5p	-0.21	0.025	0.86453723
hsa-miR-3943	-0.234	0.025	0.85027416
hsa-miR-1234-3p	-0.214	0.025	0.86214355
hsa-miR-3605-5p	-0.308	0.025	0.80776078
hsa-miR-4274	-0.177	0.025	0.88454044
hsa-miR-5588-3p	-0.206	0.025	0.86693756
hsa-miR-4445-5p	-0.234	0.025	0.85027416
hsa-miR-548k	-0.188	0.025	0.8778218
hsv2-miR-H12	-0.168	0.025	0.89007573
hsa-miR-4764-3p	1.159	0.026	2.23302592
hsa-miR-19b-3p	0.492	0.026	1.4063932
hsa-miR-582-3p	-0.209	0.026	0.86513669
hsa-miR-505-3p	-0.153	0.026	0.89937831
ebv-miR-BART20-5p	-0.192	0.026	0.87539133
hsa-miR-2117	-0.314	0.026	0.80440837
hsa-miR-512-5p	-0.139	0.026	0.90814842
hsa-miR-141-5p	-0.183	0.026	0.88086937
hsa-miR-4677-3p	0.279	0.026	1.21335356
hsa-miR-4293	-0.174	0.026	0.8863817
hsa-miR-15a-3p	-0.229	0.026	0.8532261
hsa-miR-760	-0.152	0.027	0.90000193
hsa-miR-7-1-3p	-0.199	0.027	0.87115419
hsa-miR-449c-3p	-0.216	0.027	0.86094919
hsa-miR-4286	0.266	0.027	1.20246925
hsa-miR-105-3p	-0.227	0.027	0.85440974
hsa-miR-1827	0.475	0.027	1.38991822
hsa-miR-4520a-3p	-0.189	0.027	0.87721355

hsa-miR-214-3p	0.512	0.027	1.42602572
hsa-let-7c	0.503	0.027	1.4171574
hsa-miR-4479	-0.186	0.027	0.87903956
hsa-miR-1225-5p	-0.19	0.027	0.87660572
hsv1-miR-H12	-0.218	0.027	0.85975649
hsa-miR-642b-3p	-0.237	0.027	0.8485079
hsa-miR-4526	-0.21	0.027	0.86453723
hsa-miR-1247-5p	-0.131	0.027	0.91319825
hsa-miR-4803	-0.168	0.027	0.89007573
hsa-miR-625-3p	-0.16	0.027	0.89502507
hsa-miR-5100	0.325	0.027	1.25266444
hsa-miR-574-3p	0.231	0.027	1.17364818
hsa-miR-4666a-3p	-0.193	0.027	0.87478476
hsa-miR-323b-3p	-0.178	0.027	0.88392753
hsa-let-7a-3p	0.272	0.027	1.20748059
hsa-miR-3130-5p	-0.26	0.027	0.83508792
hsa-miR-4529-5p	-0.198	0.027	0.87175824
hsa-miR-7-2-3p	-0.178	0.028	0.88392753
hsa-miR-216b	-0.178	0.028	0.88392753
hsa-miRPlus-B1114	-0.195	0.028	0.8735729
hsa-miR-208a	-0.183	0.028	0.88086937
hsa-miR-4633-3p	-0.249	0.028	0.84147948
hsa-miR-4770	-0.204	0.028	0.86814023
hsa-miR-103a-3p	0.508	0.029	1.42207741
hsa-miR-4700-3p	-0.155	0.029	0.89813237
hsa-miR-4722-5p	-0.178	0.029	0.88392753
hsa-miR-30e-3p	0.517	0.029	1.43097652
hsv1-miR-H2-5p	-0.215	0.029	0.86154616
hsa-miR-1298	-0.26	0.029	0.83508792
hsa-miR-26a-1-3p	-0.157	0.029	0.89688816
hsv2-miR-H4-5p	-0.155	0.029	0.89813237
hsa-miR-4265	-0.169	0.029	0.88945899
hsa-miR-4317	0.432	0.029	1.34910253
hsa-miR-5001-3p	-0.206	0.029	0.86693756
hsa-miR-4757-5p	-0.179	0.029	0.88331505
hsa-miR-296-3p	-0.191	0.029	0.87599832
hsa-miR-4470	-0.234	0.029	0.85027416
hsa-miR-4509	-0.307	0.029	0.80832087
hsa-miR-124-3p	-0.214	0.029	0.86214355
hsa-miR-374c-3p	-0.161	0.029	0.8944049
hsa-miR-3529-3p	-0.23	0.029	0.85263489
hsa-miR-5006-5p	-0.198	0.03	0.87175824
hsa-miR-3944-3p	-0.265	0.03	0.83219873
hsa-miR-4645-3p	-0.152	0.03	0.90000193
hsa-miR-3174	-0.146	0.03	0.90375273
hsa-miR-616-3p	-0.213	0.03	0.86274134
hsa-miR-548u	-0.237	0.03	0.8485079
hsa-miR-431-3p	-0.272	0.03	0.82817066
hsa-miR-4756-5p	-0.158	0.03	0.8962667
hsa-miR-373-3p	-0.161	0.03	0.8944049
hsa-miR-2467-5p	-0.252	0.03	0.83973149
hsa-miR-4715-5p	-0.284	0.03	0.8213107
hsa-miR-302b-3p	-0.166	0.03	0.8913105
hsa-miR-1321	0.247	0.03	1.1867368
hsa-miR-3118	-0.245	0.03	0.8438158
hsa-miR-4695-5p	-0.346	0.03	0.78676245
hsa-miR-589-3p	-0.16	0.03	0.89502507
hsa-miR-3126-3p	-0.232	0.03	0.85145371

hsa-miR-4289	0.356	0.031	1.27987241
hsa-miR-4804-3p	-0.237	0.031	0.8485079
hsa-miR-4483	-0.285	0.031	0.82074161
hsa-miR-3150b-5p	-0.192	0.031	0.87539133
hsa-miR-4640-3p	-0.202	0.031	0.86934456
hsa-miR-3150a-3p	-0.185	0.031	0.87964908
hsa-miR-1303	-0.231	0.031	0.8520441
hsa-miRPlus-G1246-3p	-0.172	0.031	0.88761134
hsa-miR-5696	-0.181	0.031	0.88209137
hsv2-miR-H6-5p	-0.305	0.031	0.80944222
hsa-miR-614	-0.146	0.031	0.90375273
hsa-miR-4491	-0.194	0.031	0.87417862
hsa-miR-4748	-0.307	0.031	0.80832087
hsa-miR-10b-5p	0.819	0.031	1.76418273
hsa-miR-4468	0.771	0.031	1.7064522
hsa-miR-654-3p	-0.144	0.031	0.90500646
hsa-miR-372	-0.192	0.031	0.87539133
hsa-miR-4797-3p	-0.184	0.031	0.88025901
hsa-miR-4635	-0.279	0.031	0.82416208
hsa-miR-369-5p	-0.145	0.031	0.90437938
hsa-miR-4691-3p	-0.168	0.031	0.89007573
hsv1-miR-H2-3p	-0.113	0.031	0.92466328
hsa-miR-4659a-5p	-0.343	0.031	0.78840017
hsa-miR-548i	-0.159	0.031	0.89564567
hsa-miR-3670	-0.192	0.032	0.87539133
hsa-miR-5007-3p	-0.174	0.032	0.8863817
hsa-miR-5094	-0.157	0.032	0.89688816
hsa-miR-561-3p	-0.125	0.032	0.91700404
hsa-miR-98-5p	0.468	0.032	1.38319063
hsa-miR-519d	-0.15	0.032	0.90125046
hsa-miR-100-3p	-0.23	0.032	0.85263489
hsa-miR-3622b-5p	-0.273	0.032	0.82759682
hsa-miR-3162-5p	-0.37	0.032	0.7737825
hsa-miR-4251	-0.18	0.033	0.882703
hsa-miR-4660	-0.149	0.033	0.90187538
ebv-miR-BART6-5p	-0.212	0.033	0.86333956
hsa-miR-92b-5p	-0.23	0.033	0.85263489
hsa-miR-4719	-0.223	0.033	0.85678195
hsa-miR-4649-3p	-0.153	0.033	0.89937831
hsa-miR-4267	-0.178	0.033	0.88392753
hsa-miR-4778-3p	0.158	0.033	1.11573932
hsa-miR-4802-3p	-0.196	0.033	0.87296759
hsa-miR-96-5p	0.269	0.033	1.20497231
hsa-miR-485-5p	-0.249	0.033	0.84147948
ebv-miR-BART18-5p	-0.252	0.033	0.83973149
hsa-miR-3938	0.559	0.033	1.47324769
hsa-miR-1231	-0.211	0.034	0.86393819
hsa-miR-5197-5p	-0.243	0.034	0.84498638
hsa-miR-27a-3p	0.963	0.034	1.94935926
hsa-miR-3126-5p	-0.178	0.034	0.88392753
hsa-miR-519a-3p	-0.141	0.034	0.90689033
hsa-miR-612	-0.22	0.034	0.85856544
hsa-miR-155-3p	-0.182	0.034	0.88148016
hsa-miR-577	-0.213	0.034	0.86274134
hsa-miR-758-3p	-0.23	0.034	0.85263489
hsa-let-7a-5p	0.839	0.034	1.7888098
hsa-miR-135b-3p	-0.219	0.034	0.85916075

hsa-miR-4790-3p	-0.29	0.034	0.81790206
hsa-let-7i-5p	0.628	0.035	1.5454211
hsa-miR-513a-3p	-0.146	0.035	0.90375273
hsa-let-7e-3p	-0.162	0.035	0.89378516
ebv-miR-BART11-3p	-0.227	0.035	0.85440974
hsa-miR-622	-0.223	0.035	0.85678195
hsa-miR-3117-3p	-0.262	0.035	0.83393104
hsa-miR-5697	-0.342	0.035	0.78894684
hsa-miR-196b-5p	0.43	0.035	1.34723358
hsa-miR-4424	-0.215	0.035	0.86154616
hsa-miR-509-5p	-0.259	0.035	0.83566696
hsa-miR-449b-3p	-0.231	0.035	0.8520441
hsa-miR-4795-5p	0.468	0.035	1.38319063
hsa-miR-1909-3p	-0.287	0.035	0.81960461
hsa-miR-3616-5p	-0.123	0.035	0.91827616
hsa-miR-587	-0.289	0.035	0.81846918
hsa-miR-146a-3p	-0.179	0.035	0.88331505
hsa-miR-130a-3p	0.736	0.035	1.66555154
hsa-miR-421	-0.174	0.035	0.8863817
hsa-miR-3134	-0.143	0.035	0.90563398
hcmv-miR-US25-1-5p	-0.227	0.035	0.85440974
hsa-miR-3668	-0.148	0.035	0.90250073
hsa-miR-374a-5p	0.431	0.036	1.34816773
hsa-miR-200c-5p	-0.225	0.036	0.85559503
hsa-miR-512-3p	-0.174	0.036	0.8863817
hsa-miR-3667-3p	-0.166	0.036	0.8913105
hsa-miR-182-5p	0.319	0.036	1.24746557
hsa-miR-382-5p	-0.156	0.036	0.89751005
hsa-miR-302e	-0.128	0.036	0.91509917
hsa-miR-520d-3p	-0.167	0.036	0.8906929
hsa-miR-181b-5p	0.206	0.036	1.15348561
hsa-miR-491-5p	-0.124	0.037	0.91763988
hsa-miR-2964a-5p	0.313	0.037	1.24228828
hsa-miR-365a-5p	-0.193	0.037	0.87478476
hsa-miR-148b-3p	0.216	0.037	1.16150873
hsa-miR-27b-3p	0.839	0.037	1.7888098
hsa-miR-1256	-0.183	0.037	0.88086937
hsa-miR-125a-5p	0.682	0.037	1.60436233
hsa-miR-4804-5p	-0.251	0.037	0.84031375
hsa-miR-4774-5p	-0.166	0.037	0.8913105
hsa-miR-3681-3p	-0.171	0.037	0.8882268
hsa-miR-4447	-0.332	0.037	0.7944344
hsa-miR-4793-5p	-0.242	0.038	0.84557229
hsa-miR-605	-0.18	0.038	0.882703
hsa-miR-4729	-0.179	0.038	0.88331505
hsa-miR-1250	-0.217	0.038	0.86035263
hsa-miR-202-5p	-0.137	0.038	0.90940825
hsa-miR-542-3p	0.354	0.038	1.27809936
hcmv-miR-US25-2-3p	-0.287	0.038	0.81960461
hsa-miR-4664-5p	-0.151	0.038	0.90062598
hsa-miR-501-5p	-0.146	0.038	0.90375273
hsa-miR-548ad	-0.169	0.038	0.88945899
hsa-miR-4652-5p	-0.195	0.038	0.8735729
hsa-miR-4796-3p	0.203	0.038	1.15108949
hsa-miR-3662	-0.168	0.038	0.89007573
hsa-miR-3675-3p	-0.197	0.038	0.87236271
hsa-miR-135b-5p	0.314	0.038	1.24314967
hsa-miR-29c-3p	0.607	0.038	1.52308874

hsa-miR-335-3p	0.334	0.038	1.26050339
hsa-miR-145-5p	0.6	0.038	1.51571657
hsa-miR-4307	-0.165	0.038	0.89192852
hsa-miR-4300	-0.165	0.038	0.89192852
ebv-miR-BART5-3p	-0.144	0.038	0.90500646
hsa-miR-1179	-0.154	0.039	0.89875513
hsa-miR-203b-3p	-0.174	0.039	0.8863817
ebv-miR-BART10-3p	-0.187	0.039	0.87843047
hsa-miR-631	-0.241	0.039	0.8461586
hsa-miR-3609	0.377	0.039	1.2986386
hsa-miR-195-5p	0.908	0.039	1.87644239
hsa-miR-450a-3p	0.169	0.039	1.12427892
hsa-miR-3913-3p	-0.157	0.039	0.89688816
hsv1-miR-H14-3p	-0.168	0.039	0.89007573
hsa-miR-34b-5p	0.718	0.039	1.64490014
hsa-miR-153	-0.161	0.039	0.8944049
hsa-let-7g-5p	0.839	0.04	1.7888098
hsa-miR-548an	0.187	0.04	1.13839403
hsa-miR-4714-3p	-0.151	0.04	0.90062598
hsa-miR-1224-3p	-0.133	0.04	0.91193317
hsa-miR-520f	-0.217	0.04	0.86035263
hsa-miR-122-5p	-0.186	0.04	0.87903956
hsa-miR-3942-5p	-0.182	0.04	0.88148016
hsa-miR-1226-3p	-0.138	0.04	0.90877812
hsa-miR-1227-3p	-0.123	0.04	0.91827616
hsa-miR-4659a-3p	-0.197	0.04	0.87236271
hsa-miR-626	-0.167	0.04	0.8906929
hsa-miR-143-5p	-0.164	0.04	0.89254697
hsa-miR-4294	-0.195	0.04	0.8735729
hsa-miR-3923	-0.164	0.04	0.89254697
hsa-miR-27a-5p	-0.139	0.041	0.90814842
ebv-miR-BART14-5p	-0.232	0.041	0.85145371
hsa-miR-548h-5p	-0.217	0.041	0.86035263
hsa-miR-186-5p	0.18	0.041	1.13288389
hsa-miR-3529-5p	-0.27	0.041	0.82931955
hsa-miR-217	-0.145	0.041	0.90437938
kshv-miR-K12-12-3p	-0.223	0.041	0.85678195
hsa-miR-145-3p	0.351	0.041	1.27544439
hsa-miR-92b-3p	0.218	0.042	1.16312004
hsa-miR-4255	-0.152	0.042	0.90000193
hsa-miR-32-3p	0.353	0.042	1.27721376
hsa-miR-519e-5p	-0.237	0.042	0.8485079
hsa-miR-4666a-5p	-0.24	0.042	0.84674531
hsa-miR-4258	-0.167	0.042	0.8906929
hsa-miR-4453	-0.148	0.042	0.90250073
mcv-miR-M1-3p	-0.156	0.042	0.89751005
hsa-miR-4797-5p	-0.404	0.042	0.75575996
hsa-miR-3918	-0.163	0.042	0.89316585
mcv-miR-M1-5p	-0.221	0.042	0.85797053
hsa-miR-517-5p	-0.179	0.042	0.88331505
hsa-miR-495-5p	0.2	0.042	1.14869835
hsa-miR-3688-5p	0.287	0.042	1.22010051
hsa-miR-609	-0.231	0.043	0.8520441
hsa-miR-92a-2-5p	-0.166	0.043	0.8913105
hsa-miR-4279	0.468	0.043	1.38319063
hsa-miR-93-3p	-0.167	0.043	0.8906929
hsa-miR-3162-3p	-0.159	0.043	0.89564567
hsa-miR-4507	-0.252	0.043	0.83973149

hsa-miR-4724-3p	-0.222	0.043	0.85737604
hsa-miR-187-3p	0.147	0.043	1.10726458
hsa-miR-3680-3p	-0.133	0.043	0.91193317
hsa-miR-5093	-0.256	0.043	0.83740649
hsa-miR-4654	-0.247	0.043	0.84264683
hsa-miR-1262	-0.237	0.043	0.8485079
hsa-miR-26b-3p	-0.121	0.043	0.91955005
hsa-miR-1185-2-3p	-0.152	0.044	0.90000193
kshv-miR-K12-7-3p	-0.162	0.044	0.89378516
hsa-miR-221-3p	0.366	0.044	1.28877463
hsa-miR-26b-5p	0.915	0.044	1.88556907
hsa-miR-885-3p	-0.212	0.044	0.86333956
hsa-miR-4495	-0.258	0.044	0.8362464
hsa-miR-1180	-0.26	0.045	0.83508792
hsa-miR-580	-0.183	0.045	0.88086937
ebv-miR-BART13-5p	-0.15	0.045	0.90125046
hsa-miR-648	-0.147	0.045	0.90312651
hsa-miR-545-3p	-0.102	0.045	0.93174043
hsa-miR-1915-3p	-0.138	0.045	0.90877812
hsv1-miR-H15	-0.239	0.045	0.84733243
hsa-miR-4513	-0.272	0.045	0.82817066
hsa-miR-4436a	-0.161	0.045	0.8944049
hsa-miR-5585-5p	-0.192	0.045	0.87539133
SNORA66	-0.171	0.046	0.8882268
hsa-miR-1249	-0.132	0.046	0.91256549
hsa-miR-4299	0.462	0.046	1.37745005
hsa-miR-4789-5p	-0.21	0.046	0.86453723
hsa-miR-633	-0.169	0.046	0.88945899
hsv1-miR-H17	-0.254	0.047	0.83856818
hsa-miR-4309	-0.157	0.047	0.89688816
hsa-miR-3926	-0.198	0.047	0.87175824
hsa-miR-4474-5p	-0.152	0.047	0.90000193
hsa-miR-3689f	-0.178	0.047	0.88392753
hsa-miR-4746-5p	-0.125	0.047	0.91700404
hsa-miR-3914	-0.208	0.047	0.86573657
hsa-miR-345-3p	-0.164	0.048	0.89254697
hsa-miR-573	-0.117	0.048	0.92210312
hsa-miR-1283	-0.21	0.048	0.86453723
hsa-miR-548p	-0.151	0.048	0.90062598
hsa-miR-3975	-0.151	0.048	0.90062598
hsa-miR-3165	-0.157	0.048	0.89688816
hsa-miR-3692-3p	-0.155	0.049	0.89813237
hsa-miR-4792	-0.266	0.049	0.8316221
hsa-miR-548aq-3p	-0.236	0.049	0.84909625
hsa-miR-644a	-0.227	0.049	0.85440974
hsa-miR-378b	-0.193	0.049	0.87478476
hsa-miR-320d	0.423	0.049	1.34071259
hsa-miR-4429	0.498	0.049	1.4122544
hsa-miR-567	-0.258	0.049	0.8362464
hsa-miR-4734	-0.253	0.049	0.83914964
hsa-miR-4438	-0.194	0.049	0.87417862
hsa-miR-885-5p	-0.184	0.049	0.88025901
hsa-miR-2114-3p	-0.192	0.049	0.87539133
hsa-miR-3683	-0.169	0.049	0.88945899
ebv-miR-BART1-5p	-0.114	0.05	0.92402257
hsa-miR-4711-3p	0.179	0.05	1.1320989
hsa-miR-3132	-0.171	0.05	0.8882268
hsa-miR-1197	-0.221	0.05	0.85797053

hsa-miR-518b	-0.146	0.05	0.90375273
hsa-miR-5571-5p	-0.177	0.05	0.88454044
hsa-miR-1307-3p	-0.155	0.05	0.89813237
hsa-miR-4701-5p	-0.143	0.05	0.90563398
hsa-miR-509-3p	-0.235	0.05	0.849685
hsa-miR-4664-3p	-0.146	0.05	0.90375273

*value of less than 0.05 is statistically significant
