# Supplementary material for: Profiling of Selected MicroRNAs in Proliferative Eutopic Endometrium of Women with Ovarian Endometriosis
Source: Biomed Res Int. 2015 Aug 20;2015:760698. doi: 10.1155/2015/760698 (PMC4558423; doi:10.1155/2015/760698)
Supplement: Supplementary file 1 — Table I: The names of miRNAs significantly differentially expressed between eutopic endometrium of patients with endometriosis vs eutopic endometrium of patients without endometriosis. Table II: The associations between miRNA and mRNA, which presents detailed results of the search of six major databases i.e targetscan, tarbase, miranda, mirbase, mirtarget2 and pictar. [file 760698.f1.zip › 760698.f1/Table II/Legend of Table II.pdf]

Legend to Tab II. supplement

Each file in this directory contains a table showing association of a particular miRNA to different mRNAs. Rows represent genes. First column shows name of a particular miRNA, the next six columns correspond to 6 databases (1 means that association was found, 0 that it was not), and the last two columns contain gene symbol and gene name, respectively.
